# Shape effect on MHD flow of time fractional Ferro-Brinkman type nanofluid with ramped heating

**DOI:** 10.1038/s41598-020-78421-z

**Published:** 2021-02-12

**Authors:** Muhammad Saqib, Ilyas Khan, Sharidan Shafie, Ahmad Qushairi Mohamad

**Affiliations:** 1 Department of Mathematical Sciences, Faculty of Science, Universiti Teknologi Malaysia JB, 81310 Johor Bahru, Johor Malaysia; 2 Department of Mathematics, College of Science Al-Zulfi, Majmaah University, Al-Majmaah, 11952 Saudi Arabia

**Keywords:** Engineering, Materials science

## Abstract

The colloidal suspension of nanometer-sized particles of Fe_3_O_4_ in traditional base fluids is referred to as Ferro-nanofluids. These fluids have many technological applications such as cell separation, drug delivery, magnetic resonance imaging, heat dissipation, damping, and dynamic sealing. Due to the massive applications of Ferro-nanofluids, the main objective of this study is to consider the MHD flow of water-based Ferro-nanofluid in the presence of thermal radiation, heat generation, and nanoparticle shape effect. The Caputo-Fabrizio time-fractional Brinkman type fluid model is utilized to demonstrate the proposed flow phenomenon with oscillating and ramped heating boundary conditions. The Laplace transform method is used to solve the model for both ramped and isothermal heating for exact solutions. The ramped and isothermal solutions are simultaneously plotted in the various figures to study the influence of pertinent flow parameters. The results revealed that the fractional parameter has a great impact on both temperature and velocity fields. In the case of ramped heating, both temperature and velocity fields decreasing with increasing fractional parameter. However, in the isothermal case, this trend reverses near the plate and gradually, ramped, and isothermal heating became alike away from the plate for the fractional parameter. Finally, the solutions for temperature and velocity fields are reduced to classical form and validated with already published results.

## Introduction

Enhanced heat transfer is significant due to its industrial and engineering applications. There are certain deficiencies in heat transfer due to the poor thermophysical properties of the working fluid. Recent advancements in nanotechnology result to develop a modern class of heat transfer fluid referred to nanofluids prepared by dispersing nanometer-sized particles 10–50 nm (nanoparticles) of metals, non-metals, and carbide in the working fluid water, oil, and alcohol, etc.^1–6^. For the first time, the term nanofluid was used by Choi and Eastman^7^. The addition of nanoparticles in traditional host fluids has the capacity to significantly improve the heat transfer rate which can be utilized in numerous areas such as, transportation industry, nuclear reactor, cooling applications, electronics, cancer therapy, and drug delivery^8^. The innovative nanofluid performs as a next generation of heat transfer fluid for novel applications in engineering and industry including aerospace, transportation, electronics, tribology, buildings, medicines. Farshad and Sheikholeslami^9^ investigated exergy loss in heat transfer in the turbulence flow of Al_2_O_3_-H_2_O through a solar collector using a finite volume method. Sadiq et al.^10^ relatively analyzed the stagnation point oscillatory flow of Cu-H_2_O and Al_2_O_3_-H_2_O micropolar nanofluid using the fifth-order R-K Fehlberg method. Alamri et al.^11^ studied heat transfer in a channel Poiseuille flow of nanofluid using Buongiorno’s nanofluids model with Stefan blowing, slip, and magnetic field effects. Ali et al.^12^ investigated the MHD flow of water-based Brinkman type nanofluid near an infinite rigid plate with variable velocity. They determined the exact analytical solutions vie the Laplace transform method. Saffarian et al.^13^ investigated the flow of Al_2_O_3_-H_2_O and CuO-H_2_O nanofluids in two U-shaped wavy pipes of the same length over a flat plate solar collector. It was indicated that CuO-H_2_O and wavy pipe enhance heat transfer rate by 78.25% and change in flow direction taken place with a higher heat transfer coefficient.

These days, the research community focuses on magnetic nanofluids (MNFs) known as Ferro-nanofluid, mainly because of its exceptional performance in the improvement of heat transfer productivity; these fluids have been utilized in numerous areas of science such as medicine, transformer cooling, nuclear fusion, and chemical engineering. The MNFs exhibits many characteristics including the controlling of thermal properties and fluid flow by means of the external magnetic field which leads to a more comprehensive thermo-magnetic convection in contrast to traditional gravitational convection. Furthermore, the MNFs are used in rods separation systems, X-rays tubes, oil lubricant bearing, sealing of hard dick damper processes. These fluids are utilized in Wi-Fi speakers, controller in electronic motors. The MNFs are considerably used in MHD based equipment such as audiometer, sensor systems, densitometer, electromechanical converter, pressure transducer, and silent printers^14^. In view of this revolutionary importance, MNF was initiated by Guptha and Guptha^15^. Many studies considered MNFs flow in different flow regimes such as Li et al.^16^ considered the flow of Ferro-nanofluid under the influence of Lorentz forces to investigate the effect of anisotropic thermal conductivity on the fluid flow and heat transfer. Shah et al.^17^ investigated micropolar Ferro-nanofluid flow over a dynamic stretching sheet in the presence of a magnetic field and thermal radiation. Kumar et al.^18^ studied the flow of hybrid Ferro-nanofluid of Fe_3_O_4_-CoFe_2_O_4_ into H_2_O-C_2_H_6_O_2_ (50–50%). It was detected that the Nusselt number of hybrid Ferro-Nanofluid is higher than Ferro-Nanofluid. Abro et al.^19^, Khan et al.^20^, Bezaatpour and Rostamzadeh^21^, Jamaluddin et al.^22^, and Aly and Ahmad^23^ focused on Ferro-nanofluid in their investigations.

The literature survey indicates that many researchers had concentrated on constant wall temperature. But in various real-world circumstances, follow variable thermal conditions at the boundary. The convection heat transfer studies are efficient to examine with step-change thermal boundary conditions. The studies with ramped wall thermal boundary conditions can be employed in thin-film photovoltaic devices to accomplish a certain finish of the system^24^. Ramped wall temperature is also necessary for heat management in buildings such as air conditioning where the constant wall thermal conditions lead to noticeable error. Motivated from the importance of step-change thermal boundary conditions (Ramped wall thermal boundary conditions), this study examines convection heat transfer with ramped boundary conditions.

To the best of the author's knowledge and from the literature survey, it is noticed that Ferro-nanofluid with a time-fractional Brinkman type fluid model with ramped heating is not reported yet. To fill the research gap, the main objective of the study is to consider the flow of Ferro-nanofluid over a vertical plate. As Ferro-nanofluid is electrically conducting thereby an external magnetic field is employed normal to the flow direction. The Caputo-Fabrizio fractional operator^25^ is used to fractionalized the Brinkman type fluid model. As Ali et al.^26^ utilized the Caputo-Fabrizio fractional derivative to investigate the MHD flow simultaneously with heat and mass transfer in Walters’-B fluid in the present magnetic field and porous medium. Khan et al.^27–29^ analyzed the flow of Casson and H_2_O-CNTs nanofluid in a microchannel using a fractional derivatives approach. They determine the exact solutions by using the Laplace transform method. In a similar way, the proposed model is solved for exact analytical solutions vie the Laplace transform method. These solutions are presented for temperature and velocity fields for both ramped and isothermal heating. The ramped and isothermal solutions are simultaneously plotted in the various figures to study the influence of embedded flow parameters with the physical explanation. Eventually, by making $\alpha \to 1$, the classical solutions are recovered for temperature and velocity field for both ramped and isothermal heating from these solutions and validated with previously published work.

## Description of the problem

**Figure 1:**
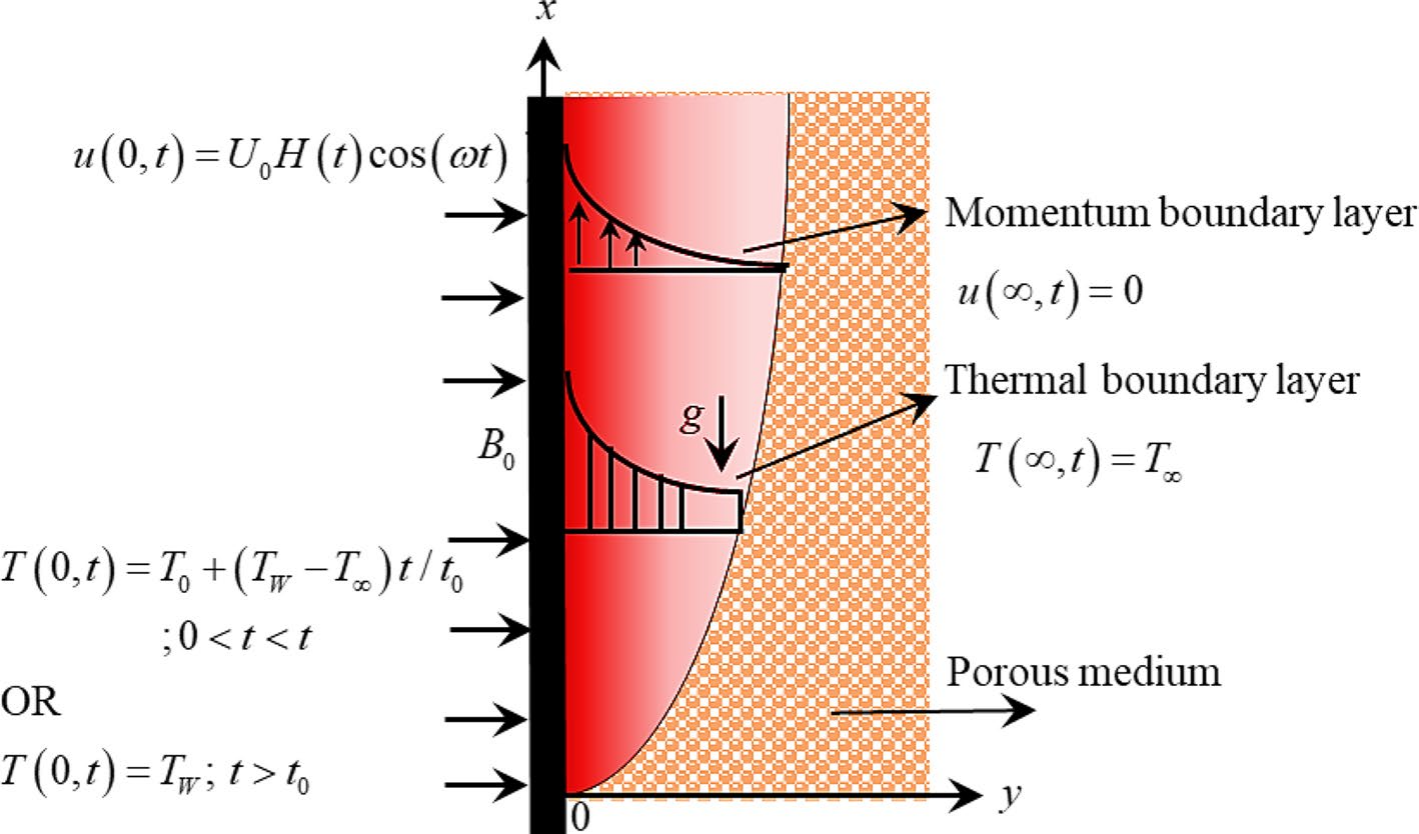
Physical sketch and coordinate system.

Assume heat transfer in MHD flow of time-fractional Ferro-Brinkman type nanofluid near an infinite vertical plate along the $x$-axis and $y$-axis is selected transverse to it. Magnetic nanoparticles of different shapes (blade, brick, spherical, and platelet) are dispersed into the water as base fluid to form Ferro-Brinkman type nanofluid. Thermal radiation, heat generation, and ramped wall heating are also considered. At $t \le 0$, the fluid and plate are set in rest with $u\left( {y,0} \right) = 0$ and $T\left( {y,0} \right) = T_{\infty }$ where $T_{\infty }$ is the ambient temperature. Afterward at $t = 0^{ + }$, the velocity and temperature fields are switched to $u\left( {0,t} \right) = U_{0} H\left( t \right)\cos \left( {\omega t} \right)$ and $T\left( {0,t} \right) = T_{0} + \left( {T_{W} - T_{\infty } } \right)t/t_{0}$ if $0 < t < t_{0}$ or $T\left( {0,t} \right) = T_{W}$ if $t > t_{0}$ respectively. At this phase, the fluid starts flowing in $x$-direction as presented in Fig. 1. The Ferro-Brinkman type nanofluid experience magnetic force because the fluid is electrically conducting, thereby, an external magnetic field is employed normally to the flow direction. The governing equations of the proposed model are derived in the following section.

## Mathematese formulation

In accordance with Rajagopal^30^ and Fetecau, Fetecau^31^, the linear momentum equation for Brinkman type fluid can be written as

1 $$ \rho \frac{{D{\text{V}}}}{Dt} = \nabla \cdot {\underline {\text{T}}} + \rho {\text{F}} - {\text{I}}_{0} , $$

where $\frac{D}{Dt} = \frac{\partial }{\partial t} + u\frac{\partial }{\partial x} + v\frac{\partial }{\partial y} + w\frac{\partial }{\partial z}$ refers to material time derivatives, ${\text{V}}$ is the velocity vector, $\underline {\text{T}}$ represents the Cauchy stress tensor, $\rho {\text{F}}$ depicts the body forces, and ${\text{I}}_{0}$ exhibits the interaction force of porous medium which can be expressed as

2 $$ {\text{I}}_{0} = \alpha_{d} {\text{V}}, $$

where $\alpha_{d}$ is a positive coefficient of drag force which yields Eq. (1) to the following form

3 $$ \rho \left( {\frac{{\partial {\text{V}}}}{\partial t} + \left( {{\text{V}}.\nabla } \right){\text{V}}} \right) = \nabla .{\underline {\text{T}}} + \rho {\text{F}} - \alpha_{d} {\text{V}}. $$

In the case of Brinkman type fluid, the constitutive equation of Cauchy stress tensor is expressed by^32^

4 $$ {\underline {{\text{T}}}} =  - p\underline {\text{I}} + \mu {\underline {\text{A}}}_{1} , $$

where $p$ is the scalar pressure, $\underline {\text{I}}$ is the identity tensor, $\mu$ is the dynamic viscosity, and ${\underline {\text{A}}}_{1}$ is the Rivlin–Ericksen tensor determined by

5 $$ {\underline {\text{A}}}_{{1}} = \nabla {\text{V}} + \left( {\nabla {\text{V}}} \right)^{T} , $$

where the superscript $T$ refers to the matrix transpose and $\nabla {\text{V}}$ represents the gradient of the velocity. In the case of the proposed problems, the unsteady, incompressible, unidirectional, and one-dimensional flow is considered thereby, the velocity vector is defined as

6 $$ {\text{V}} = \left( {u\left( {y,t} \right),0,0} \right) = u\left( {y,t} \right){\text{i}}. $$

Bearing in mind, Eqs. (4) and (6), the $\nabla \cdot {{\underline{\text{T} }}}$ is determined as

7 $$ \nabla \cdot {{\underline{\text {T} }}} =  - \frac{\partial p}{{\partial x}} + \mu \frac{{\partial^{2} u\left( {y,t} \right)}}{{\partial y^{2} }}, $$

whereas, the fluid flow is considered in $x$-direction, therefore, $p \ne p\left( {y,z} \right)$ which lead $\partial p/\partial y = 0$ $\partial p/\partial z = 0$. Introducing Eq. (7) into Eq. (3) and bearing in mind Eq. (6) which yield to

8 $$ \rho \frac{{\partial u\left( {y,t} \right)}}{\partial t} = - \frac{\partial p}{{\partial x}} + \mu \frac{{\partial^{2} u\left( {y,t} \right)}}{{\partial y^{2} }} + \rho {\text{F}} - \alpha_{d} u\left( {y,t} \right). $$

Based on Jaluria^33^, the body forces $\rho {\text{F}}$ for convection flow of electrically conducting Brinkman type fluid is given by

9 $$ \rho {\text{F}} = {\text{J}} \times {\text{B}} + \rho {\text{g}}, $$

where ${\text{J}} \times {\text{B}}$ is the Lorentz force, ${\text{J}}$ is the current density, ${\text{B}} = {\text{B}}_{0} + {\text{b}}$ is the magnetic flux intensity, ${\text{B}}_{0}$ is applied magnetics filed acting in $y$-direction, ${\text{b}}$ is the induced magnetic field and ${\text{g}} = \left( { - g,0,0} \right)$ is the gravitation acceleration. The Lorentz force can be defined by Maxwell’s set of equations as^34^

10 $$ \left. \begin{gathered} \nabla .{\text{B}} = 0, \hfill \\ \nabla \times {\text{B}} = \mu_{m} {\text{J}}, \hfill \\ \nabla \times {\text{E}} = - \frac{{\partial {\text{B}}}}{\partial t}, \hfill \\ \end{gathered} \right\}, $$

where $\mu_{m}$ is the magnetic permeability and ${\text{E}}$ is electric field intensity. The current density ${\text{J}}$ is described by the generalized Ohm’s law as^35^

11 $$ {\text{J}} = \sigma \left( {{\text{E}} + {\text{V}} \times {\text{B}}} \right), $$

where $\sigma$ is the electrical conductivity. Meanwhile, the magnetic Reynolds number is assumed small enough so that the induced field ${\text{b}}$ is neglected compared to the applied field ${\text{B}}_{0}$. Furthermore, it is assumed that there is no polarization and applied voltages thereby, the electric field ${\text{E}}$ is ignored. Hence, Eq. (11) takes the following form

12 $$ {\text{J}} = \sigma \left( {{\text{V}} \times {\text{B}}_{0} } \right) = \sigma \left| {\begin{array}{*{20}c} {\text{i}} & {\text{j}} & {\text{k}} \\ u & 0 & 0 \\ 0 & {B_{0} } & 0 \\ \end{array} } \right| = \left( {0,0,\sigma B_{0} u\left( {y,t} \right)} \right). $$

Keeping in mind Eq. (12), the Lorentz force ${\text{J}} \times {\text{B}}$ became

13 $$ {\text{J}} \times {\text{B}} = \left| {\begin{array}{*{20}c} {\text{i}} & {\text{j}} & {\text{k}} \\ 0 & 0 & {\sigma B_{0} u} \\ 0 & {B_{0} } & 0 \\ \end{array} } \right| = \left( { - \sigma B_{0}^{2} u\left( {y,t} \right),0,0} \right). $$

Introducing Eq. (13) into Eq. (9) and then into Eq. (8) which gives the following

14 $$ \rho \frac{\partial u}{{\partial t}} = - \frac{\partial p}{{\partial x}} + \mu \frac{{\partial^{2} u\left( {y,t} \right)}}{{\partial y^{2} }} - \sigma B_{0}^{2} u\left( {y,t} \right) + \alpha_{d} u\left( {y,t} \right) - \rho g. $$

Referred to Jaluria^33^, the pressure $p$ in Eq. (14) can be written in the following form

15 $$ p = p_{h} + p_{d} , $$

where $p_{h}$ is the hydrostatic pressure $p_{d}$ is the dynamic pressure. The proposed problem is considered for convection heat transfer therefore, $p_{d}$.can be neglected. According to White^36^, the hydrostatic pressure $p_{h}$ in the case of convection heat transfer can be written as

16 $$ \frac{{\partial p_{h} }}{\partial x} = - \rho_{\infty } g, $$

where $\rho_{\infty }$ is the ambient density of the fluid. Introducing Eq. (16) into Eq. (14) yield to

17 $$ \rho \frac{\partial u}{{\partial t}} = \mu \frac{{\partial^{2} u\left( {y,t} \right)}}{{\partial y^{2} }} - \sigma B_{0}^{2} u + \alpha_{d} u + \left( {\rho_{\infty } - \rho } \right)g. $$

Assuming that $\beta_{T}$ is the volumetric thermal expansion of the fluid then according to Boussinesq’s approximation^33^, $\beta_{T}$ can be written as

18 $$ \beta_{T} = - \frac{1}{\rho }\left( {\frac{\partial \rho }{{\partial T}}} \right)_{p} \approx - \frac{1}{\rho }\frac{\Delta \rho }{{\Delta T}} = - \frac{1}{\rho }\frac{{\rho_{\infty } - \rho }}{{T_{\infty } - T\left( {y,t} \right)}}, $$

or

19 $$ \rho \beta_{T} \left( {T\left( {y,t} \right) - T_{\infty } } \right) = \rho_{\infty } - \rho . $$

Introducing Eq. (19) into Eq. (17) yield to

20 $$ \rho \frac{\partial u}{{\partial t}} = \mu \frac{{\partial^{2} u\left( {y,t} \right)}}{{\partial y^{2} }} - \sigma B_{0}^{2} u\left( {y,t} \right) + \alpha_{d} u\left( {y,t} \right) + g\rho \beta_{T} \left( {T\left( {y,t} \right) - T_{\infty } } \right), $$

or

21 $$ \frac{{\partial u\left( {y,t} \right)}}{\partial t} = \upsilon \frac{{\partial^{2} u\left( {y,t} \right)}}{{\partial y^{2} }} - \frac{{\sigma B_{0}^{2} u\left( {y,t} \right)}}{\rho } - \beta^{*} u\left( {y,t} \right) + g\beta_{T} \left( {T\left( {y,t} \right) - T_{\infty } } \right), $$

where $\beta^{*} = \alpha_{d} /\rho$ is the Brinkman type fluid parameter which corresponds to the drag force of highly non-Darcy’s porous medium. The energy equation together with thermal radiation and heat generation is given by^37^

22 $$ \rho C_{p} \frac{{\partial T\left( {y,t} \right)}}{\partial t} = k\frac{{\partial^{2} T\left( {y,t} \right)}}{{\partial y^{2} }} - \frac{{\partial q_{r} }}{\partial y} + Q_{0} \left( {T\left( {y,t} \right) - T_{\infty } } \right). $$

The radiative heat flux $q_{r}$ in Eq. (22) is formulated by using via the Roseland approximation as^38^

23 $$ q_{r} = - \frac{{4\sigma_{1} }}{{3k_{1} }}\frac{{\partial T^{4} }}{\partial y}. $$

The $T^{4}$ is expanded along $T_{\infty }$ by using the Taylor series as

24 $$ T^{4} = T_{\infty }^{4} + \frac{{4T_{\infty }^{3} }}{1!}\left( {T - T_{\infty } } \right) + \frac{{12T_{\infty }^{2} }}{2!}\left( {T - T_{\infty } } \right)^{2} + \frac{{24T_{\infty } }}{3!}\left( {T - T_{\infty } } \right)^{3} \cdots $$

The temperature gradient is assumed to be small enough so, the higher-order terms are neglected which yield to

25 $$ T^{4} \approx T_{\infty }^{4} + \frac{{4T_{\infty }^{3} }}{1!}\left( {T - T_{\infty } } \right). $$

The further simplification of Eq. (25) yield to the following

26 $$ T^{4} \approx 4T_{\infty }^{3} T - 3T_{\infty }^{4} . $$

The simplified form of $T^{4}$ from Eq. (26), is used in Eq. (23) which yield to

27 $$ q_{r} = - \frac{{16\sigma_{1} T_{\infty }^{3} }}{{3k_{1} }}\frac{\partial T}{{\partial y}}. $$

Differentiating Eq. (27) with respect to “$y$” yield to the following

28 $$ \frac{{\partial q_{r} }}{\partial y} = - \frac{{16\sigma_{1} T_{\infty }^{3} }}{{3k_{1} }}\frac{{\partial^{2} T}}{{\partial y^{2} }}. $$

Incorporating Eq. (28) into Eq. (22) yield to the following

29 $$ \left( {\rho C_{p} } \right)\frac{{\partial T\left( {y,t} \right)}}{\partial t} = k\left( {1 + \frac{{16\sigma_{1} T_{\infty }^{3} }}{{3k_{1} k}}} \right)\frac{{\partial^{2} T\left( {y,t} \right)}}{{\partial y^{2} }} + Q_{0} \left( {T\left( {y,t} \right) - T_{\infty } } \right). $$

For enhanced heat transfer, the Fe_3_O_4_ has been dispersed into the water as base fluid to form water-Ferro-nanofluid. As refer to Khanafar et al.^39^ and Tiwari and Das^40^ Eqs. (21 and (29) can be written for Ferro-nanofluid flow as

30 $$ \rho_{nf} \left( {\frac{{\partial u\left( {y,t} \right)}}{\partial t} + \beta^{*} u\left( {y,t} \right)} \right) = \mu_{nf} \frac{{\partial^{2} u\left( {y,t} \right)}}{{\partial y^{2} }} - \sigma_{nf} B_{0}^{2} u\left( {y,t} \right) + g\left( {\rho \beta_{T} } \right)_{nf} \left( {T\left( {y,t} \right) - T_{\infty } } \right), $$

31 $$ \left( {\rho C_{p} } \right)_{nf} \frac{{\partial T\left( {y,t} \right)}}{\partial t} = k_{nf} \left( {1 + \frac{{16\sigma_{1} T_{\infty }^{3} }}{{3k_{1} k_{nf} }}} \right)\frac{{\partial^{2} T\left( {y,t} \right)}}{{\partial y^{2} }} + Q_{0} \left( {T\left( {y,t} \right) - T_{\infty } } \right), $$

where $\rho_{nf}$ is the density, $u\left( {y,t} \right)$ is the velocity, $\beta^{*}$ is the Brinkman type fluid parameter, $\mu_{nf}$ is the dynamic velocity, $\sigma_{nf}$ is the electrical conductivity, $B_{0}$ is the uniform magnetic field, $g$ gravitational acceleration, $\left( {\beta_{T} } \right)_{nf}$ is the thermal expansion, $T\left( {y,t} \right)$ is the temperature, $\left( {C_{p} } \right)_{nf}$ is the heat capacitance, $k_{nf}$ is the thermal conductivity, $q_{r}$ is the radiative heat flux and $Q_{0}$ is the heat generation. The corresponding initial and boundary conditions are given as

32 $$ u\left( {y,0} \right) = 0,\,T\left( {y,0} \right) = T_{\infty } ,\,\forall y \le 0, $$

33 $$ \left. \begin{gathered} u\left( {0,t} \right) = U_{0} H\left( t \right)\cos \left( {\omega t} \right);\,\quad \,\forall t \ge 0^{ + } , \hfill \\ T\left( {0,t} \right) = \left\{ \begin{gathered} T_{0} + \left( {T_{W} - T_{\infty } } \right)\frac{t}{{t_{0} }};\,\,\quad {\text{if}}\,0 < t < t_{0} , \hfill \\ T_{W} \,\,\,\,\,\,\,\,\,\,\,\,\,\,\,\,\,\,\,\,\,\,\,\,\,\,\,\,\,;\,\,\quad {\text{if}}\,t > t_{0} \hfill \\ \end{gathered} \right. \hfill \\ u\left( {y,t} \right) \to 0\,\,{\text{and}}\,\,T\left( {y,t} \right) \to T_{\infty } ;\,\quad {\text{if}}\,y \to \infty , \hfill \\ \end{gathered} \right\}. $$

The terms $\rho_{nf}$, $\mu_{nf}$, $\sigma_{nf}$, $\left( {\beta_{T} } \right)_{nf}$, $\left( {C_{p} } \right)_{nf}$ and $k_{nf}$ appeared in Eqs. (30) and (31) for the enhanced thermophysical properties nanofluid with different shapes (blade, brick, spherical, and platelet) nanoparticles defined as^41^.

34 $$ \rho_{nf} = \left( {1 - \phi } \right)\rho_{f} + \phi \rho_{s} , $$

35 $$ \mu_{nf} = \mu_{f} \left( {1 + a\phi + b\phi^{2} } \right), $$

36 $$ \frac{{\sigma_{nf} }}{{\sigma_{f} }} = 1 + \frac{{3\left( {\frac{{\sigma_{s} }}{{\sigma_{f} }} - 1} \right)\phi }}{{\left( {\frac{{\sigma_{s} }}{{\sigma_{f} }} + 2} \right) - \left( {\frac{{\sigma_{s} }}{{\sigma_{f} }} - 1} \right)\phi }}, $$

37 $$ \left( {\rho \beta_{T} } \right)_{nf} = \left( {1 - \phi } \right)\left( {\rho \beta_{T} } \right)_{f} + \phi \left( {\rho \beta_{T} } \right)_{s} , $$

38 $$ \left( {\rho C_{p} } \right)_{nf} = \left( {1 - \phi } \right)\left( {\rho C_{p} } \right)_{f} + \phi \left( {\rho C_{p} } \right)_{s} , $$

and

39 $$ \,K_{nf} = K_{f} \left[ {\frac{{K_{s} + \left( {n - 1} \right)K_{f} + \left( {n - 1} \right)\left( {K_{s} - K_{f} } \right)\phi }}{{K_{s} + \left( {n - 1} \right)K_{f} - \left( {K_{s} - K_{f} } \right)\phi }}} \right], $$

where the subscript $nf$ is used for nanofluid, $f$ for base fluid water, and $s$ for solid nanoparticles Fe_3_O_4_. Furthermore, in Eq. (35) $a$ and $b$ correspond to shape constant which affects the density factor of nanofluid and in Eq. (39) $n$ is the experimental shape constituent. $n$ can be evaluated as

40 $$ n = \frac{3}{\psi }, $$

where $\psi$ is the sphericity of nanoparticles which influences the thermal conductivity. The different shape nanoparticles and the corresponding values of $a$, $b$ and $\psi$ are presented in Table 1^42,43^

**Table 1:** Different shapes of nanoparticles with corresponding values of $a$, $b$ and $\psi$.

Nanoparticle type	Sketch	$a$	$b$	$\psi$
Blade		14.6	123.3	0.36
Brick		1.9	471.4	0.81
Cylindrical		13.5	909.4	0.62
Platelet		37.1	612.6	0.52

In this study magnetic nanoparticle (Fe_3_O_4_) of different shapes is dissolved in water (H_2_O) as base fluid to form magnetic nanofluid (MNF). The physical values of the thermal properties of nanoparticles and base are given in Table 1^44,45^

## Solutions of the problem

This section presents the exact solutions for the magnetic flow of time-fractional Ferro-Brinkman type nanofluid under the effect of a normal magnetic field. In this section, the problem modeled in Sect. 3 is first transformed to dimensionless form to diminish the units for simplification and reduction of number variables. For this purpose, the following dimensionless variables

$$ u^{*} = \frac{u}{{U_{0} }},\,y^{*} = \frac{{U_{0} }}{{\upsilon_{f} }}y,\,t^{*} = \frac{t}{{t_{0} }},\,t_{0} = \frac{{\upsilon_{f} }}{{U_{0}^{2} }},\,\theta = \frac{{T - T_{\infty } }}{{T_{W} - T_{\infty } }} $$

are implemented into Eqs. (30)–(33) after dropping the * sign for simplicity yield to the following form

41 $$ \phi_{0} \left\{ {\frac{{\partial u\left( {y,t} \right)}}{\partial t} + \beta u\left( {y,t} \right)} \right\} = \phi_{1} \frac{{\partial^{2} u\left( {y,t} \right)}}{{\partial y^{2} }} - \phi_{2} Mu\left( {y,t} \right) + \phi_{3} Gr\theta \left( {y,t} \right), $$

42 $$ \phi_{4} \frac{{\partial \theta \left( {y,t} \right)}}{\partial t} = \frac{{\phi_{5} }}{{\Pr_{eff} }}\frac{{\partial^{2} \theta \left( {y,t} \right)}}{{\partial y^{2} }} + Q\theta \left( {y,t} \right), $$

together with the following dimensionless conditions

43 $$ u\left( {y,0} \right) = 0,\,\theta \left( {y,0} \right) = 0,\,\forall y \le 0, $$

44 $$ \left. \begin{gathered} u\left( {0,t} \right) = H\left( t \right)\cos \left( {\omega t} \right)\,\,{\text{or}}\,\,\sin \left( {\omega t} \right);\,\quad \,\forall t \ge 0^{ + } \hfill \\ T\left( {0,t} \right) = \left\{ \begin{gathered} t\,\,;\quad {\text{if}}\,0 < t < 1 \hfill \\ 1\,\,;\,\quad \,{\text{if}}\,t > 1 \hfill \\ \end{gathered} \right. \hfill \\ u\left( {y,t} \right) \to 0\,\quad and\quad \,T\left( {y,t} \right) \to 0;\quad \,{\text{if}}\,y \to \infty \hfill \\ \end{gathered} \right\}, $$

and

$$\begin{gathered} \beta = \frac{{U_{0}^{2} \beta^{*} }}{{\upsilon_{f} }},\quad M = \frac{{\upsilon_{f} \sigma_{f} B_{0}^{2} }}{{U_{0}^{2} \rho_{f} }},\quad Gr = \frac{{g\left( {\beta_{T} \upsilon } \right)_{f} \left( {T_{W} - T_{\infty } } \right)}}{{U_{0}^{3} }},\quad \Pr = \left( {\frac{{\mu C_{p} }}{k}} \right)_{f} ,\quad Nr = \frac{{16\sigma_{1} T_{\infty }^{3} }}{{3k_{1} k_{f} }},\quad \Pr_{eff} = \frac{{\phi_{5} \Pr }}{{\phi_{5} + Nr}}, \hfill \\ Q = \frac{{\upsilon_{f} Q_{0} }}{{U_{0}^{2} \left( {\rho Cp} \right)_{f} }},\quad \phi_{0} = \left( {1 - \phi } \right) + \phi \frac{{\rho_{s} }}{{\rho_{f} }},\quad \phi_{1} = 1 + a\phi + b\phi^{2} ,\quad \phi_{2} = \frac{{\sigma_{nf} }}{{\sigma_{f} }},\quad \phi_{3} = \left( {1 - \phi } \right) + \phi \frac{{\left( {\rho \beta_{T} } \right)_{s} }}{{\left( {\rho \beta_{T} } \right)_{f} }}, \hfill \\ \phi_{4} = \left( {1 - \phi } \right) + \phi \frac{{\left( {\rho C_{p} } \right)_{s} }}{{\left( {\rho C_{p} } \right)_{f} }},\quad \phi_{5} = \frac{{k_{nf} }}{{k_{f} }} \hfill \\ \hfill \\ \end{gathered}$$

where $\beta$ is the Brinkman parameter, $M$ is the magnetic number, $Gr$ is thermal Grashof number, $\Pr$ is the Prandtl number, $Nr$ is the radiation parameter, $\Pr_{eff}$ is the effective Prandtl number, $Q$ is the heat generation parameter, and $\phi_{0}$, $\phi_{1}$, $\phi_{2}$, $\phi_{3}$, $\phi_{4}$, $\phi_{5}$ are constant terms. The Caputo–Fabrizio time-fractional derivative is used to transform Eqs. (41) and (42) to time-fractional for as

45 $$ \phi_{0} \left\{ {\mathcal{D}_{t}^{\alpha } u\left( {y,t} \right) + \beta u\left( {y,t} \right)} \right\} = \phi_{1} \frac{{\partial^{2} u\left( {y,t} \right)}}{{\partial y^{2} }} - \phi_{2} Mu\left( {y,t} \right) + \phi_{3} Gr\theta \left( {y,t} \right), $$

46 $$ \phi_{4} \mathcal{D}_{t}^{\alpha } \theta \left( {y,t} \right) = \frac{{\phi_{5} }}{{\Pr_{eff} }}\frac{{\partial^{2} \theta \left( {y,t} \right)}}{{\partial y^{2} }} + Q\theta \left( {y,t} \right). $$

The Caputo–Fabrizio time-fractional operator $\mathcal{D}_{t}^{\alpha } \left( {,.,} \right)$ appeared in Eqs. (45) and (46) is defined by^25^

47 $$ \mathcal{D}_{t}^{\alpha } f\left( {y,t} \right) = \frac{N\left( \alpha \right)}{{1 - \alpha }}\int\limits_{0}^{t} {\exp \left( { - \frac{{\alpha \left( {t - \tau } \right)}}{1 - \alpha }} \right)} \frac{{\partial f\left( {y,\tau } \right)}}{\partial \tau }d\tau \,\,;\,0 < \alpha < 1, $$

where $N\left( \alpha \right)$ is the normalization function with the following property

48 $$ N\left( 1 \right) = N\left( 0 \right) = 1. $$

Using Eq. (48) the Laplace transform of Eq. (47) is given by

49 $$ \mathcal{L}\left\{ {\mathcal{D}_{t}^{\alpha } f\left( {y,t} \right)} \right\}\left( q \right) = \frac{{q\overline{f}\left( {y,q} \right) - f\left( {y,0} \right)}}{{\left( {1 - \alpha } \right)q + \alpha }},\,\,0 < \alpha < 1, $$

which be reduced for integer-order time derivative as

50 $$ \begin{aligned} \mathop {\lim }\limits_{\alpha \to 1} \left[ {\mathcal{L}\left\{ {\mathcal{D}_{t}^{\alpha } f\left( {y,t} \right)} \right\}\left( q \right)} \right] & = \mathop {\lim }\limits_{\alpha \to 1} \left\{ {\frac{{q\overline{f}\left( {y,q} \right) - f\left( {y,0} \right)}}{{\left( {1 - \alpha } \right)q + \alpha }}} \right\} \\ & = q\overline{f}\left( {y,q} \right) - f\left( {y,0} \right) = \mathcal{L}\left\{ {\frac{{\partial f\left( {y,t} \right)}}{\partial t}} \right\}. \\ \end{aligned} $$

### Solution for temperature filed

This presents the solutions for temperature filed in both ramped and isothermal heating case.

#### Solutions for temperature filed with ramped heating

In order to solve the energy equation for the temperature field, the Laplace transform is employed to Eq. (46), keeping in view Eq. (49) and using initial condition from Eq. (43) which yield to

51 $$ \frac{{q\overline{\theta }\left( {y,q} \right) - \theta \left( {y,0} \right)}}{{\left( {1 - \alpha } \right)q + \alpha }} = \frac{{\phi_{5} }}{{\phi_{4} \Pr_{eff} }}\frac{{d^{2} \overline{\theta }\left( {y,q} \right)}}{{dy^{2} }} + Q\overline{\theta }\left( {y,q} \right), $$

which gives the following on further simplification

52 $$ \frac{{d\overline{\theta }\left( {y,q} \right)}}{{dy^{2} }} - \frac{{a_{1} q - a_{2} }}{{q + b_{1} }}\overline{\theta }\left( {y,q} \right) = 0, $$

along with the transformed corresponding boundary conditions

53 $$ \left. {\overline{\theta }\left( {0,q} \right) = \int\limits_{0}^{1} {t \cdot e^{ - qt} + \int\limits_{1}^{\infty } {1 \cdot e^{ - qt} } = \frac{{1 - e^{ - q} }}{{q^{2} }}} \,{\text{and}}\,\overline{\theta }\left( {\infty ,q} \right) = 0} \right\}, $$

where

$$ a_{1} = a_{0} b_{0} - Q_{1} ,a_{2} = Q_{1} b_{1} ,\,a_{0} = \frac{{\phi_{4} \Pr_{eff} }}{{\phi_{5} }},\,Q_{1} = \frac{{Q\phi_{4} \Pr_{eff} }}{{\phi_{5} }},\,b_{0} = \frac{1}{1 - \alpha },\,b_{1} = b_{0} \alpha . $$

The exact analytical solutions of Eq. (52) can be determined by using the transform boundary conditions for Eq. (53) as

54 $$ \overline{\theta }\left( {y,q} \right) = \frac{1}{{q^{2} }}e^{{ - y\sqrt {\frac{{a_{1} q - a_{2} }}{{q + b_{1} }}} }} - e^{ - q} \frac{1}{{q^{2} }}e^{{ - y\sqrt {\frac{{a_{1} q - a_{2} }}{{q + b_{1} }}} }} , $$

which correspond to the solutions of temperature field for ramped heating in the Laplace transform domain. Equation (52) can be further simplified as

55 $$ \overline{\theta }\left( {y,q} \right) = \overline{\theta }_{Ramp} \left( {y,q} \right) - e^{ - q} \overline{\theta }_{Ramp} \left( {y,q} \right), $$

where

56 $$ \overline{\theta }_{Ramp} \left( {y,q} \right) = \frac{1}{{q^{2} }}e^{{ - y\sqrt {\frac{{a_{1} q - a_{2} }}{{q + b_{1} }}} }} . $$

The inverse Laplace transform is used to invert back Eq. (55) to the time domain as

57 $$ \theta \left( {y,t} \right) = \theta_{Ramp} \left( {y,t} \right) - \theta_{Ramp} \left( {y,t - 1} \right)H\left( {t - 1} \right), $$

where $H\left( {t - 1} \right)$ is the Heaviside unit step function and the term $\theta_{Ramp} \left( {y,t} \right)$ is defined by

58 $$ \theta_{Ramp} \left( {y,t} \right) = te^{{ - y\sqrt {a_{1} } }} - \int\limits_{0}^{\infty } {\int\limits_{0}^{t} {\left( {t - \tau } \right)} } \frac{{y\sqrt {p_{1} } }}{{2s\sqrt {\pi \tau } }}e^{{ - b_{1} \tau - \frac{{y^{2} }}{4s} - a_{1} s}} I_{1} \left( {2\sqrt {p_{1} s\tau } } \right)dsd\tau , $$

where

$$ p_{1} = - a_{2} - a_{1} b_{1} . $$

#### Solutions for temperature filed with isothermal heating

In order to find exact solutions for isothermal heating, the boundary condition in the Laplace transform domain is given by

59 $$ \overline{\theta }\left( {0,q} \right) = \frac{1}{q}, $$

The exact analytical solutions of Eq. (52) is obtained by using Eq. (59) as

60 $$ \overline{\theta }\left( {y,q} \right) = \frac{1}{q}e^{{ - y\sqrt {\frac{{a_{1} q - a_{2} }}{{q + b_{1} }}} }} , $$

The final exact solution for isothermal heating is obtained after applying the inverse Laplace transform to Eq. (60) which yield to

61 $$ \theta \left( {y,t} \right) = e^{{ - y\sqrt {a_{1} } }} - \int\limits_{0}^{\infty } {\int\limits_{0}^{t} {\frac{{y\sqrt {p_{1} } }}{{2s\sqrt {\pi \tau } }}e^{{ - b_{1} \tau - \frac{{y^{2} }}{4s} - a_{1} s}} I_{1} \left( {2\sqrt {p_{1} s\tau } } \right)dsd\tau } } . $$

The exact solutions corresponding ramped and isothermal heating are respectively depicted in Eqs. (57) and (61) which satisfy the impose conditions in both cases. The exact solutions for the velocity field corresponding to ramped and the isothermal heating is presented in the following section.

### Solution for velocity field

This section presents exact analytical solutions for the velocity field for both ramped and isothermal heating.

#### Solutions for velocity field with ramped heating

The Laplace transform is applied to Eq. (45) using initial condition from Eq. (43) yield to

62 $$ \phi_{0} \left\{ {\frac{{q\overline{u}\left( {y,q} \right) - u\left( {y,0} \right)}}{{\left( {1 - \alpha } \right)q + \alpha }} + \beta \overline{u}\left( {q,t} \right)} \right\} = \phi_{1} \frac{{d^{2} \overline{u}\left( {y,q} \right)}}{{dy^{2} }} - \phi_{2} M\overline{u}\left( {q,t} \right) + \phi_{3} Gr\overline{\theta }\left( {q,t} \right), $$

which takes the following form after simplification

63 $$ \frac{{d^{2} \overline{u}\left( {y,q} \right)}}{{dy^{2} }} - \left( {\frac{{a_{3} q + a_{4} }}{{q + b_{1} }}} \right)\overline{u}\left( {y,q} \right) = - Gr_{0} \left( {\frac{{1 - e^{ - q} }}{{q^{2} }}} \right)e^{{ - y\sqrt {\frac{{a_{1} q - a_{2} }}{{q + b_{1} }}} }} , $$

along with the transformed velocity boundary conditions

64 $$ \overline{u}\left( {0,q} \right) = \frac{q}{{q^{2} + \omega^{2} }}\,\,{\text{and}}\,\,\overline{u}\left( {\infty ,q} \right) = 0, $$

where

$$ a_{3} = \frac{1}{{\phi_{1} }}\left( {\phi_{0} b_{0} + \phi_{0} \beta + \phi_{2} M} \right),\,\,a_{4} = \frac{1}{{\phi_{1} }}\left( {\phi_{0} \beta b_{1} + \phi_{2} Mb_{1} } \right),\,\,Gr_{0} = \frac{{\phi_{3} }}{{\phi_{1} }}Gr. $$

The analytical solutions of Eq. (63) can be obtained by using the boundary conditions from Eq. (64) as

65 $$ \overline{u}\left( {y,q} \right) = \frac{q}{{q^{2} + \omega^{2} }}e^{{ - y\sqrt {\frac{{a_{3} q + a_{4} }}{{q + b_{1} }}} }} + \left( {\frac{{Gr_{0} \left( {q + b_{1} } \right)}}{{a_{5} q - a_{6} }}} \right)\left( {\frac{{1 - e^{ - q} }}{{q^{2} }}} \right)\left( {e^{{ - y\sqrt {\frac{{a_{3} q + a_{4} }}{{q + b_{1} }}} }} - e^{{ - y\sqrt {\frac{{a_{1} q - a_{2} }}{{q + b_{1} }}} }} } \right), $$

where

$$ a_{5} = a_{1} - a_{3} ,\,\,a_{6} = a_{2} + a_{4} . $$

In order to find the inverse Laplace transform, Eq. (65) can be written in a more suitable form as

66 $$ \begin{gathered} \overline{u}\left( {y,q} \right) = \overline{u}_{c} \left( {y,q} \right) + \overline{u}_{1} \left( q \right)\left\{ {\overline{u}_{{2\left( {Ramp} \right)}} \left( {y,q} \right) - e^{ - q} \overline{u}_{{2\left( {Ramp} \right)}} \left( {y,q} \right)} \right\} \hfill \\ \,\,\,\,\,\,\,\,\,\,\,\, - \overline{u}_{1} \left( {y,q} \right)\overline{\theta }\left( {y,q} \right), \hfill \\ \end{gathered} $$

where

67 $$ \overline{u}_{c} \left( {y,q} \right) = \frac{q}{{q^{2} + \omega^{2} }}e^{{ - y\sqrt {\frac{{a_{3} q + a_{4} }}{{q + b_{1} }}} }} , $$

68 $$ \overline{u}_{1} \left( q \right) = \frac{{Gr_{0} \left( {q + b_{1} } \right)}}{{a_{5} q - a_{6} }}, $$

69 $$ \overline{u}_{{2\left( {Ramp} \right)}} \left( {y,q} \right) = \frac{1}{{q^{2} }}e^{{ - y\sqrt {\frac{{a_{3} q + a_{4} }}{{q + b_{1} }}} }} , $$

and $\overline{\theta }\left( {y,q} \right)$ is previously defined by Eq. (56). Now, the inverse Laplace transform is applied to Eq. (66) which gives

70 $$ \begin{aligned} u\left( {y,t} \right) & = u_{c} \left( {y,t} \right) + u_{1} \left( t \right)*\left\{ {u_{{2\left( {Ramp} \right)}} \left( {y,t} \right) - H\left( {t - 1} \right)u_{{2\left( {Ramp} \right)}} \left( {y,t - 1} \right)} \right\} \\ & \quad - u_{1} \left( {y,t} \right)*\theta \left( {y,t} \right), \\ \end{aligned} $$

where

71 $$ \overline{u}_{c} \left( {y,q} \right) = te^{{ - y\sqrt {a_{1} } }} - \int\limits_{0}^{\infty } {\int\limits_{0}^{t} {\cos \left( {t - \tau } \right)} } \frac{{y\sqrt {p_{2} } }}{{2s\sqrt {\pi \tau } }}e^{{ - b_{1} \tau - \frac{{y^{2} }}{4s} - a_{1} s}} I_{1} \left( {2\sqrt {p_{2} s\tau } } \right)dsd\tau , $$

72 $$ u_{1} \left( t \right) = \left( {a_{7} e^{{\frac{{a_{6} }}{{a_{5} }}t}} + \frac{1}{{a_{5} }}\delta \left( t \right)} \right), $$

73 $$ u_{2(Ramp)} \left( {y,t} \right) = te^{{ - y\sqrt {a_{1} } }} - \int\limits_{0}^{\infty } {\int\limits_{0}^{t} {\left( {t - \tau } \right)} } \frac{{y\sqrt {p_{2} } }}{{2s\sqrt {\pi \tau } }}e^{{ - b_{1} \tau - \frac{{y^{2} }}{4s} - a_{1} s}} I_{1} \left( {2\sqrt {p_{2} s\tau } } \right)dsd\tau , $$

and

$$ p_{2} = a_{4} - a_{3} b_{1} ,\,a_{7} = Gr_{0} \left( {\frac{{a_{6} + a_{5} b_{1} }}{{a_{5}^{2} }}} \right). $$

The symbol * presents the convolutions product and $\theta \left( {y,t} \right)$ is depicted in Eq. (57). It is worth highlighting here that Eq. (70) characterize the exact solutions for the velocity field with ramped heating.

#### Solutions for velocity field with isothermal heating

Next, Eq. (45) is solved again for isothermal heating as

74 $$ \overline{u}_{Iso} \left( {y,q} \right) = \frac{q}{{q^{2} + \omega^{2} }}e^{{ - y\sqrt {\frac{{a_{3} q + a_{4} }}{{q + b_{1} }}} }} + \left( {\frac{{Gr_{0} \left( {q + b_{1} } \right)}}{{a_{3} q - a_{2} }}} \right)\left( \frac{1}{q} \right)\left( {e^{{ - y\sqrt {\frac{{a_{3} q + a_{4} }}{{q + b_{1} }}} }} - e^{{ - y\sqrt {\frac{{a_{1}q-{a_{2}}}}{{q + b_{1} }}} }} } \right), $$

For convenience in inverse Laplace transform, Eq. (74) can be written in more in suitable form as

75 $$ \begin{gathered} \overline{u}\left( {y,q} \right) = \overline{u}_{c} \left( {y,q} \right) + \overline{u}_{1} \left( q \right)\left\{ {\overline{u}_{{3\left( {Iso} \right)}} \left( {y,q} \right) - e^{ - q} \overline{u}_{{3\left( {Iso} \right)}} \left( {y,q} \right)} \right\} \hfill \\ \,\,\,\,\,\,\,\,\,\,\,\, - \overline{u}_{1} \left( {y,q} \right)\overline{\theta }\left( {y,q} \right), \hfill \\ \end{gathered} $$

where in this case, terms $\overline{\theta }\left( {y,q} \right)$,$\overline{u}_{c} \left( {y,q} \right)$, $\overline{u}_{1} \left( q \right)$, and are already defined in Eqs. (60), (67), and (68) respectively. The term $\overline{u}_{{3\left( {Iso} \right)}} \left( {y,q} \right)$ newly appeared is presented by

76 $$ \overline{u}_{{3\left( {Iso} \right)}} \left( {y,q} \right) = \frac{1}{q}e^{{ - y\sqrt {\frac{{a_{1} q + a_{2} }}{{q + b_{1} }}} }} . $$

The solution for isothermal heating is obtained by taking the inverse Laplace transform of Eq. (75) which yield to

77 $$ \begin{aligned} u\left( {y,t} \right) & = u_{C} \left( {y,t} \right) + u_{1} \left( t \right)*\left\{ {u_{{3\left( {Iso} \right)}} \left( {y,t} \right) - H\left( {t - 1} \right)u_{{3\left( {Iso} \right)}} \left( {y,t - 1} \right)} \right\} \\ & \quad \, - u_{1} \left( {y,t} \right)*\theta \left( {y,t} \right), \\ \end{aligned} $$

where

78 $$ u_{{3\left( {Iso} \right)}} \left( {y,t} \right) = e^{{ - y\sqrt {a_{1} } }} - \int\limits_{0}^{\infty } {\int\limits_{0}^{t} {\frac{{y\sqrt {p_{2} } }}{{2s\sqrt {\pi \tau } }}e^{{ - b_{1} \tau - \frac{{y^{2} }}{4s} - a_{1} s}} I_{1} \left( {2\sqrt {p_{2} s\tau } } \right)dsd\tau } } , $$

and in this case, the terms $\theta \left( {y,t} \right)$ $u_{c} \left( {y,t} \right)$, $u_{1} \left( t \right)$ are previously defined in Eqs. (61), (71) and (72) respectively. This completes the solutions for the proposed problem.

## Limiting cases

This section presents the limiting solutions by making $\alpha \to 1$ in Eqs. (57), (61), (70) and (77), for both velocity and temperature fields.

### Limiting solutions for temperature

This subsection highlights limiting solutions for ramped and isothermal heating for the temperature field.

#### Limiting solution for temperature filed with ramped heating

Keeping in mind Eq. (50), employing $\lim_{\alpha \to 1}$ to Eq. (51) which yield to

79 $$ q\overline{\theta }\left( {y,q} \right) - \theta \left( {y,0} \right) = \frac{{\phi_{5} }}{{\phi_{4} \Pr_{eff} }}\frac{{d^{2} \overline{\theta }\left( {y,q} \right)}}{{dy^{2} }} + Q\overline{\theta }\left( {y,q} \right), $$

where $\overline{\theta }\left( {y,q} \right)$ is the classical temperature in the Laplace transform domain and $\theta \left( {y,0} \right)$ is the initial condition. After using the boundary conditions from Eq. (53), the analytical solution of Eq. (79) is given by

80 $$ \overline{\theta }\left( {y,q} \right) = \frac{1}{{q^{2} }}e^{{ - y\sqrt {a_{0} q - Q_{1} } }} - e^{ - q} \frac{1}{{q^{2} }}e^{{ - y\sqrt {a_{0} q - Q_{1} } }} , $$

which can be written as

81 $$ \overline{\theta }\left( {y,q} \right) = \overline{\Psi }\left( {y\sqrt {a_{0} } ,\frac{{Q_{1} }}{{a_{0} }},q} \right) - e^{ - q} \overline{\Psi }\left( {y\sqrt {a_{0} } ,\frac{{Q_{1} }}{{a_{0} }},q} \right), $$

where

82 $$ \overline{\Psi }\left( {y\sqrt {a_{0} } ,\frac{{Q_{1} }}{{a_{0} }},q} \right) = \frac{1}{{q^{2} }}e^{{ - y\sqrt {a_{0} } \sqrt {q - \frac{{Q_{1} }}{{a_{0} }}} }} , $$

Upon inverting the Laplace transform, Eq. (81) yield to

83 $$ \theta \left( {y,t} \right) = \Psi \left( {y\sqrt {a_{0} } ,\frac{{Q_{1} }}{{a_{0} }},t} \right) - \Psi \left( {y\sqrt {a_{0} } ,\frac{{Q_{1} }}{{a_{0} }},t - 1} \right)H\left( {t - 1} \right), $$

where

84 $$ \Psi \left( {y\sqrt {a_{0} } ,\frac{{Q_{1} }}{{a_{0} }},t} \right) = \frac{1}{2}\left\{ \begin{gathered} e^{{y\sqrt {Q_{1} } }} erfc\left( {\frac{{y\sqrt {a_{0} } }}{2\sqrt t } + \frac{{Q_{1} }}{{a_{0} }}\sqrt t } \right)\left( {t + \frac{{ya_{0} \sqrt {a_{0} } }}{{2Q_{1} }}} \right) \hfill \\ + e^{{ - y\sqrt {Q_{1} } }} erfc\left( {\frac{{y\sqrt {a_{0} } }}{2\sqrt t } - \frac{{Q_{1} }}{{a_{0} }}\sqrt t } \right)\left( {t - \frac{{ya_{0} \sqrt {a_{0} } }}{{2Q_{1} }}} \right) \hfill \\ \end{gathered} \right\}. $$

#### Limiting solution for temperature field with isothermal heating

To find the classical solution for temperature field in case of isothermal heating, Eq. (79) is analytically solved by using the boundary condition Eq. (59) which gives

85 $$ \overline{\theta }\left( {y,q} \right) = \overline{\Theta }\left( {y\sqrt {a_{0} } ,\frac{{Q_{1} }}{{a_{0} }},q} \right) = \frac{1}{q}e^{{ - y\sqrt {a_{0} } \sqrt {q - \frac{{Q_{1} }}{{a_{0} }}} }} , $$

The inverse Laplace transform is employed to Eq. (85) which yield to

86 $$ \theta \left( {y,t} \right) = \Theta \left( {y\sqrt {a_{0} } ,\frac{{Q_{1} }}{{a_{0} }},t} \right) = \frac{1}{2}\left\{ {e^{{ - y\sqrt {Q_{1} } }} erfc\left( {\frac{{y\sqrt {a_{0} } }}{2\sqrt t } - \sqrt {\frac{{Q_{1} }}{{a_{0} }}t} } \right) + e^{{y\sqrt {Q_{1} } }} erfc\left( {\frac{{y\sqrt {a_{0} } }}{2\sqrt t } + \sqrt {\frac{{Q_{1} }}{{a_{0} }}t} } \right)} \right\}. $$

**Figure 2:**
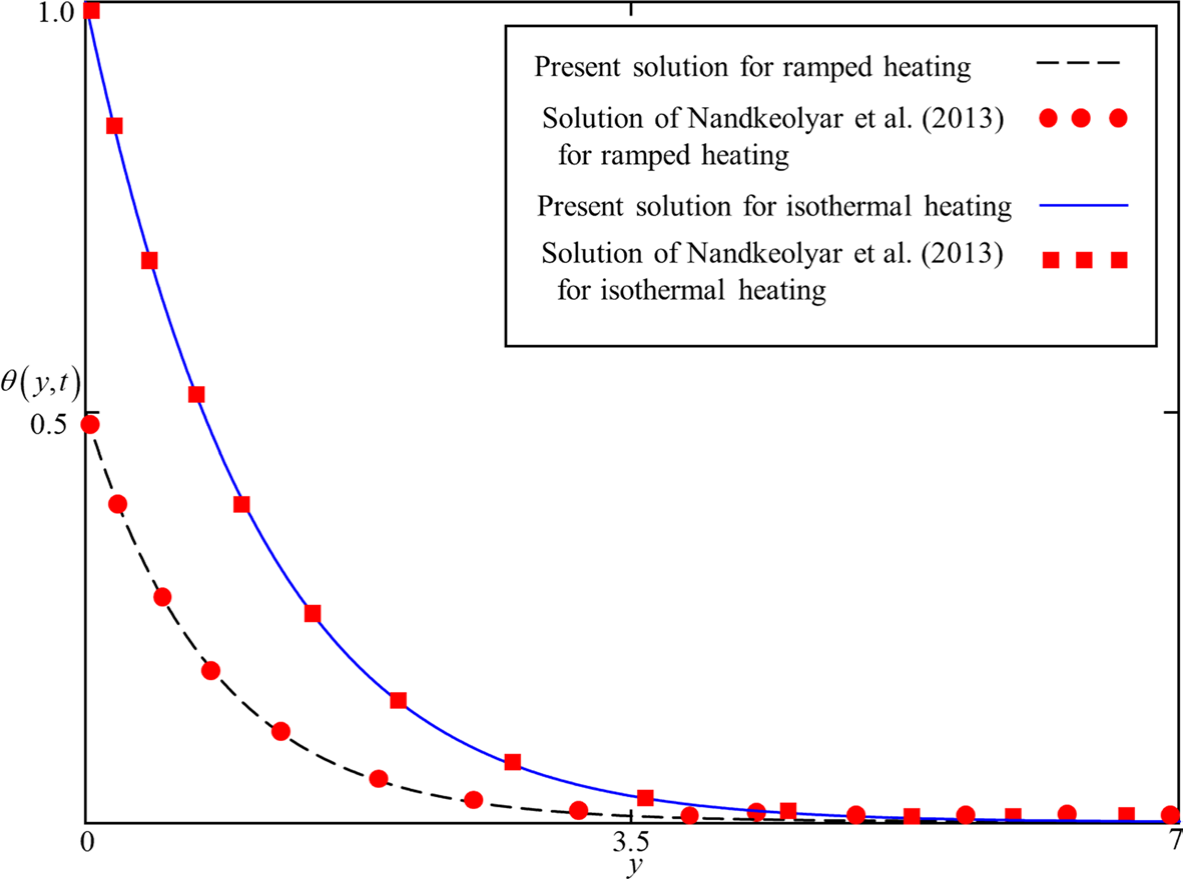
Comparison of present solutions presented in Eqs. (83), (86) and Nandkeolyar et al. .^45^, Eq. (19) for $t = 0.5,\,1.5$.

It is worth mentioning here that by assuming the thermal conductivity of Maxwell from^41^ and using numerical values of thermophysical properties of TiO_2_ nanoparticles From Table 2, the solutions obtained in Eq. (83), can be reduced by setting $Q \to 0$, to that of Nandkeolyar et al.^45^ (Eq. 19). In the absence of heat generation, the solutions presented in Eqs. (83) and (86) from the present study and Eq. (19) from Nandkeolyar et al.^45^, for $t = 0.5,\,1.5$ are computed and displayed in Fig. 2. This figure clearly indicates that the solutions are identical which validates the present solutions for the Temperature field.

**Table 2:** Physical values of thermal properties of nanoparticles and base fluid.

Material	Base fluid	Nanoparticles	
	H_2_O	F_3_O_4_	TiO
$\rho \,\,\,\left( {{{{\text{kg}}} \mathord{\left/ {\vphantom {{{\text{kg}}} {{\text{m}}^{{3}} }}} \right. \kern-\nulldelimiterspace} {{\text{m}}^{{3}} }}} \right)$	997.1	5200	425
$C_{p} \,\,\left( {{{\text{J}} \mathord{\left/ {\vphantom {{\text{J}} {{\text{kg}}\,{\text{K}}}}} \right. \kern-\nulldelimiterspace} {{\text{kg}}\,{\text{K}}}}} \right)$	4179	670	6862
$k\,\,\left( {{{\text{W}} \mathord{\left/ {\vphantom {{\text{W}} {{\text{m}}\,{\text{K}}}}} \right. \kern-\nulldelimiterspace} {{\text{m}}\,{\text{K}}}}} \right)$	0.613	6	8.9538
$\beta_{T} \times 10^{ - 5} \,\,({\text{K}}^{ - 1} )$	21	1.3	0.9
$\sigma$	0.05	25,000	$1 \times 10^{ - 12}$
Pr	6.2	–	–

### Limiting solution for velocity field

The limiting solutions for the velocity field in case of ramped and isothermal heating is introduced in this subsection.

#### Limiting solution for velocity field with ramped heating

While taking into account Eqs. (50), (62) is reduced to classical form by applying $\lim_{\alpha \to 1}$ to which yield to

87 $$ \phi_{0} \left\{ {q\overline{u}\left( {y,q} \right) - u\left( {y,0} \right) + \beta \overline{u}\left( {q,t} \right)} \right\} = \phi_{1} \frac{{d^{2} \overline{u}\left( {y,q} \right)}}{{dy^{2} }} - \phi_{2} M\overline{u}\left( {q,t} \right) + \phi_{3} Gr\overline{\theta }\left( {q,t} \right). $$

The analytical solution of Eq. (87) is determined by using Eq. (80) and boundary conditions from Eq. (64) as

88 $$ \overline{u}\left( {y,q} \right) = \left\{ {\frac{q}{{q^{2} + \omega^{2} }} + \frac{{Gr_{0} }}{{a_{8} q + a_{9} }}\left( {\frac{{1 - e^{ - q} }}{{q^{2} }}} \right)} \right\}e^{{ - y\sqrt {a_{6} q + a_{7} } }} - \frac{{Gr_{0} }}{{a_{8} q + a_{9} }}\left( {\frac{{1 - e^{ - q} }}{{q^{2} }}} \right)e^{{ - y\sqrt {a_{0} q + Q_{1} } }} . $$

where $$a_{s} = a_{0} - a_{6}, \; \; a_{9} = a_{7} + Q_{1}$$.

By using partial fraction, Eq. (88) can be reduced to the following form

89 $$ \begin{aligned} \overline{u}\left( {y,q} \right) & = \frac{1}{{2\left( {q + i\omega } \right)}}e^{{ - y\sqrt {a_{6} q + a_{7} } }} + \frac{1}{{2\left( {q - i\omega } \right)}}e^{{ - y\sqrt {a_{6} q + a_{7} } }} \\ & \quad + \frac{{Gr_{0} }}{{a_{8} q + a_{9} }}\left( {\frac{1}{{q^{2} }}e^{{ - y\sqrt {a_{6} q + a_{7} } }} - e^{ - q} \frac{1}{{q^{2} }}e^{{ - y\sqrt {a_{6} q + a_{7} } }} } \right) \\ & \quad - \frac{{Gr_{0} }}{{a_{8} q + a_{9} }}\left( {\frac{1}{{q^{2} }}e^{{ - y\sqrt {a_{0} q + Q_{1} } }} - e^{ - q} \frac{1}{{q^{2} }}e^{{ - y\sqrt {a_{0} q + Q_{1} } }} } \right). \\ \end{aligned} $$

In order to find the inverse Laplace transform, Eq. (89) can be written in a more suitable form as

90 $$ \begin{aligned} \overline{u}\left( {y,q} \right) & = \frac{1}{2}\overline{\Phi }\left( {y\sqrt {a_{6} } ,\frac{{a_{7} }}{{a_{6} }}, - i\omega ,q} \right) + \frac{1}{2}\overline{\Phi }\left( {y\sqrt {a_{6} } ,\frac{{a_{7} }}{{a_{6} }},i\omega ,q} \right) \\ & \quad + \overline{F}\left( q \right)\left\{ {\overline{\Psi }\left( {y\sqrt {a_{6} } ,\,\frac{{a_{7} }}{{a_{6} }},q} \right) - e^{ - q} \overline{\Psi }\left( {y\sqrt {a_{6} } ,\,\frac{{a_{7} }}{{a_{6} }},q} \right)} \right\} \\ & \quad - \overline{F}\left( q \right)\left\{ {\overline{\Psi }\left( {y\sqrt {a_{0} } ,\frac{{Q_{1} }}{{a_{0} }},q} \right) - e^{ - q} \overline{\Psi }\left( {y\sqrt {a_{0} } ,\frac{{Q_{1} }}{{a_{0} }},q} \right)} \right\}, \\ \end{aligned} $$

where

91 $$ \overline{\Phi }\left( {y\sqrt {a_{6} } ,\frac{{a_{7} }}{{a_{6} }}, - i\omega ,q} \right) = \frac{1}{{\left( {q + i\omega } \right)}}e^{{ - y\sqrt {a_{6} q + a_{7} } }} , $$

92 $$ \overline{\Phi }\left( {y\sqrt {a_{6} } ,\frac{{a_{7} }}{{a_{6} }},i\omega ,q} \right) = \frac{1}{{\left( {q - i\omega } \right)}}e^{{ - y\sqrt {a_{6} q + a_{7} } }} , $$

93 $$ \overline{\Psi }\left( {y\sqrt {a_{6} } ,\,\frac{{a_{7} }}{{a_{6} }},q} \right) = \frac{1}{{q^{2} }}e^{{ - y\sqrt {a_{6} q + a_{7} } }} , $$

94 $$ \overline{F}\left( q \right) = \frac{{Gr_{0} }}{{a_{8} q + a_{9} }}, $$

and the function $\overline{\Psi }\left( {y\sqrt {a_{0} } ,\frac{{Q_{1} }}{{a_{0} }},q} \right)$ is previously defined in Eq. (82). Applying the inverse Laplace transform to Eq. (90) which yield to

95 $$ \begin{aligned} u\left( {y,t} \right) & = \frac{1}{2}\Phi \left( {y\sqrt {a_{6} } ,\frac{{a_{7} }}{{a_{6} }}, - i\omega ,t} \right) + \frac{1}{2}\Phi \left( {y\sqrt {a_{6} } ,\frac{{a_{7} }}{{a_{6} }},i\omega ,t} \right) \\ & \quad + F\left( q \right)*\left\{ {\Psi \left( {y\sqrt {a_{6} } ,\,\frac{{a_{7} }}{{a_{6} }},t} \right) - e^{ - q} \Psi \left( {y\sqrt {a_{6} } ,\,\frac{{a_{7} }}{{a_{6} }},t - 1} \right)H\left( {t - 1} \right)} \right\} \\ & \quad - F\left( q \right)*\left\{ {\Psi \left( {y\sqrt {a_{0} } ,\frac{{Q_{1} }}{{a_{0} }},t} \right) - \Psi \left( {y\sqrt {a_{0} } ,\frac{{Q_{1} }}{{a_{0} }},t - 1} \right)H\left( {t - 1} \right)} \right\}, \\ \end{aligned} $$

where

96 $$ \begin{aligned} \Phi \left( {y\sqrt {a_{6} } ,\frac{{a_{7} }}{{a_{6} }}, - i\omega ,t} \right) & = \frac{{e^{i\omega t} }}{2}\left\{ \begin{gathered} e^{{ - y\sqrt {a_{6} } \sqrt {\frac{{a_{7} }}{{a_{6} }} - i\omega } }} erfc\left( {\frac{{y\sqrt {a_{6} } }}{2\sqrt t } - \sqrt {\left( {\frac{{a_{7} }}{{a_{6} }} - i\omega } \right)t} } \right) \hfill \\ e^{{y\sqrt {a_{6} } \sqrt {\frac{{a_{7} }}{{a_{6} }} - i\omega } }} erfc\left( {\frac{{y\sqrt {a_{6} } }}{2\sqrt t } + \sqrt {\left( {\frac{{a_{7} }}{{a_{6} }} - i\omega } \right)t} } \right) \hfill \\ \end{gathered} \right\}, \\ \Phi \left( {y\sqrt {a_{6} } ,\frac{{a_{7} }}{{a_{6} }},i\omega ,t} \right) & = \frac{{e^{i\omega t} }}{2}\left\{ \begin{gathered} e^{{ - y\sqrt {a_{6} } \sqrt {i\omega + \frac{{a_{7} }}{{a_{6} }}} }} erfc\left( {\frac{{y\sqrt {a_{6} } }}{2\sqrt t } - \sqrt {\left( {i\omega + \frac{{a_{7} }}{{a_{6} }}} \right)t} } \right) \hfill \\ e^{{y\sqrt {a_{6} } \sqrt {\frac{{a_{7} }}{{a_{6} }} - i\omega } }} erfc\left( {\frac{{y\sqrt {a_{6} } }}{2\sqrt t } + \sqrt {\left( {i\omega + \frac{{a_{7} }}{{a_{6} }}} \right)t} } \right) \hfill \\ \end{gathered} \right\}, \\ \Psi \left( {y\sqrt {a_{6} } ,\,\frac{{a_{7} }}{{a_{6} }},t} \right) & = \frac{1}{2}\left\{ \begin{gathered} e^{{y\sqrt {a_{7} } }} erfc\left( {\frac{{y\sqrt {a_{6} } }}{2\sqrt t } + \frac{{a_{7} }}{{a_{6} }}\sqrt t } \right)\left( {t + \frac{{ya_{6} \sqrt {a_{6} } }}{{2a_{7} }}} \right) \hfill \\ + e^{{ - y\sqrt {a_{7} } }} erfc\left( {\frac{{y\sqrt {a_{6} } }}{2\sqrt t } - \frac{{a_{7} }}{{a_{6} }}\sqrt t } \right)\left( {t - \frac{{ya_{6} \sqrt {a_{6} } }}{{2a_{7} }}} \right) \hfill \\ \end{gathered} \right\}, \\ F\left( q \right) & = \frac{{Gr_{0} }}{{a_{8} }}e^{{ - \frac{{a_{9} }}{{a_{8} }}t}} , \\ \end{aligned} $$

and the function $\Psi \left( {y\sqrt {a_{0} } ,\frac{{Q_{1} }}{{a_{0} }},t} \right)$ is already presented in Eq. (84) .

#### Limiting solution for velocity field with isothermal heating

In order to reduce Eq. (62) to classical form for the velocity field with isothermal heating, Eq. (85) is used which yield to

97 $$ \overline{u}\left( {y,q} \right) = \left\{ {\frac{q}{{q^{2} + \omega^{2} }} + \frac{1}{q}\left( {\frac{{Gr_{0} }}{{a_{8} q + a_{9} }}} \right)} \right\}e^{{ - y\sqrt {a_{6} q + a_{7} } }} - \frac{1}{q}\left( {\frac{{Gr_{0} }}{{a_{8} q + a_{9} }}} \right)e^{{ - y\sqrt {a_{0} q + Q_{1} } }} , $$

For the convenience in the inverse Laplace transform, Eq. (97) is reduced to the following form

98 $$ \begin{aligned} \overline{u}\left( {y,q} \right) & = \frac{1}{{2\left( {q + i\omega } \right)}}e^{{ - y\sqrt {a_{6} q + a_{7} } }} + \frac{1}{{2\left( {q - i\omega } \right)}}e^{{ - y\sqrt {a_{6} q + a_{7} } }} \\ & \quad + \frac{{Gr_{0} }}{{a_{8} }}\frac{1}{q}\left( {\frac{1}{{q + \frac{{a_{9} }}{{a_{8} }}}}} \right)e^{{ - y\sqrt {a_{6} q + a_{7} } }} - \frac{{Gr_{0} }}{{a_{8} }}\frac{1}{q}\left( {\frac{1}{{q + \frac{{a_{9} }}{{a_{8} }}}}} \right)e^{{ - y\sqrt {a_{0} q + Q_{1} } }} , \\ \end{aligned} $$

which can be written in a further simplified form as

99 $$ \begin{gathered} \overline{u}\left( {y,q} \right) = \frac{1}{2}\overline{\Phi }\left( {y\sqrt {a_{6} } ,\frac{{a_{7} }}{{a_{6} }}, - i\omega ,q} \right) + \frac{1}{2}\overline{\Phi }\left( {y\sqrt {a_{6} } ,\frac{{a_{7} }}{{a_{6} }},i\omega ,q} \right) \hfill \\ + \frac{{Gr_{0} }}{{a_{8} }}\frac{1}{q}\overline{\Phi }\left( {y\sqrt {a_{6} } ,\frac{{a_{7} }}{{a_{6} }},\frac{{a_{9} }}{{a_{8} }},q} \right) - \frac{{Gr_{0} }}{{a_{8} }}\frac{1}{q}\overline{\Phi }\left( {y\sqrt {a_{0} } ,\frac{{Q_{1} }}{{a_{0} }},\frac{{a_{9} }}{{a_{8} }},q} \right), \hfill \\ \end{gathered} $$

where

100 $$ \overline{\Phi }\left( {y\sqrt {a_{6} } ,\frac{{a_{7} }}{{a_{6} }},\frac{{a_{9} }}{{a_{8} }},q} \right) = \frac{1}{{q + \frac{{a_{9} }}{{a_{8} }}}}e^{{ - y\sqrt {a_{6} q + a_{7} } }} , $$

101 $$ \overline{\Phi }\left( {y\sqrt {a_{0} } ,\frac{{Q_{1} }}{{a_{0} }},\frac{{a_{9} }}{{a_{8} }},q} \right) = \frac{1}{{q + \frac{{a_{9} }}{{a_{8} }}}}e^{{ - y\sqrt {a_{0} q + Q_{1} } }} , $$

and functions $\overline{\Phi }\left( {y\sqrt {a_{6} } ,\frac{{a_{7} }}{{a_{6} }}, - i\omega ,q} \right)$ and $\overline{\Phi }\left( {y\sqrt {a_{6} } ,\frac{{a_{7} }}{{a_{6} }},i\omega ,q} \right)$ are given in Eqs. (91) and (92) respectively. Next, taking the inverse Laplace transform of Eq. (99), the exact solution for the classical velocity field in case of isothermal heating is given by

102 $$ \begin{aligned} u\left( {y,t} \right) & = \frac{1}{2}\Phi \left( {y\sqrt {a_{6} } ,\frac{{a_{7} }}{{a_{6} }}, - i\omega ,t} \right) + \frac{1}{2}\Phi \left( {y\sqrt {a_{6} } ,\frac{{a_{7} }}{{a_{6} }},i\omega ,t} \right) \\ & \quad + \frac{{Gr_{0} }}{{a_{8} }}\int\limits_{0}^{t} {\Phi \left( {y\sqrt {a_{6} } ,\frac{{a_{7} }}{{a_{6} }},\frac{{a_{9} }}{{a_{8} }},\tau } \right)d\tau } - \frac{{Gr_{0} }}{{a_{8} }}\int\limits_{0}^{t} {\Phi \left( {y\sqrt {a_{0} } ,\frac{{Q_{1} }}{{a_{0} }},\frac{{a_{9} }}{{a_{8} }},\tau } \right)d\tau } , \\ \end{aligned} $$

**Figure 3:**
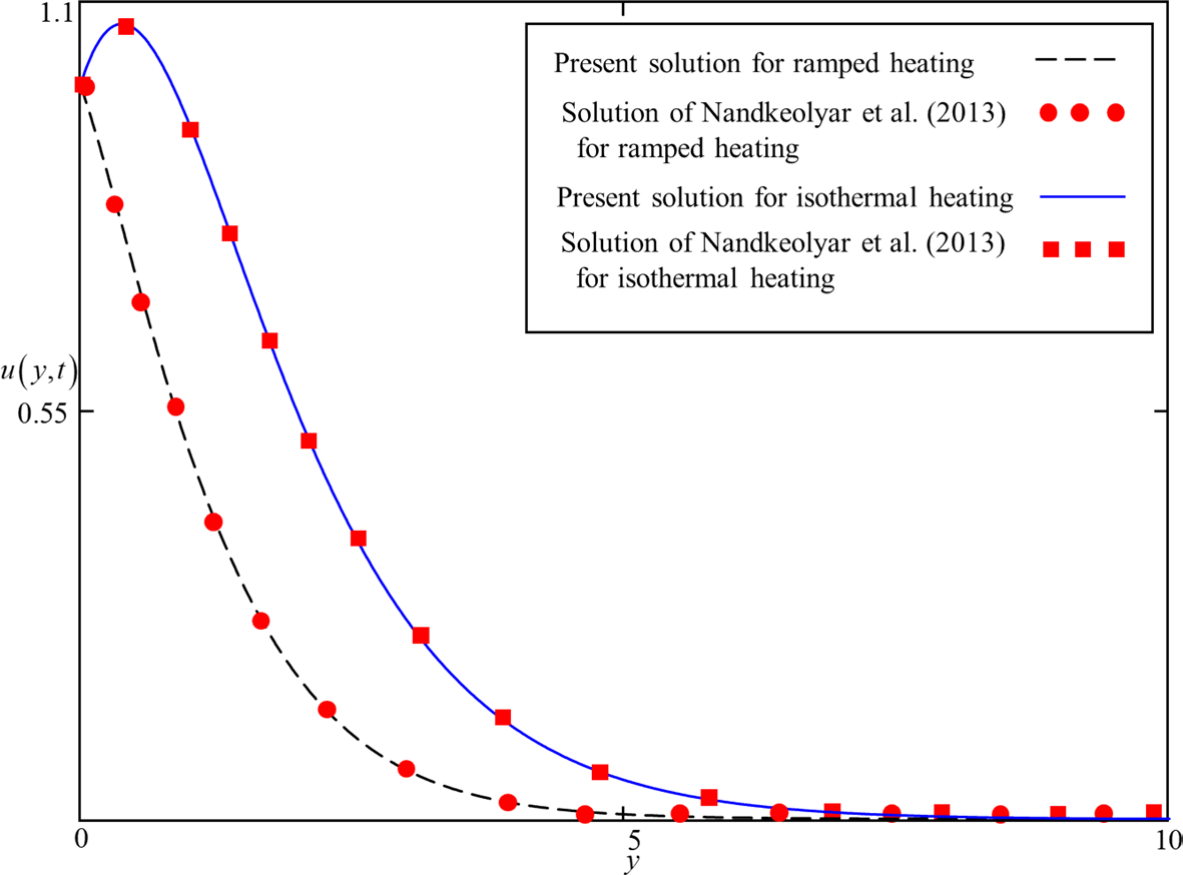
Comparison of present solutions presented in Eqs. (95), (102) and Nandkeolyar et al.^45^, Eq. (20) for $t = 0.5,\,1.5$

Besides this, the viscosity of Brinkman and thermal conductivity of Maxwell’s from^41^ are set in Eqs. (95) and (102). After making $\beta \to 0$ and $Q \to 0$, in Eqs. (95) and (102) with TiO_2_ nanoparticles are computed for $t = 0.5,\,1.5$ together with Eq. (20) from Nandkeolyar et al.^45^ and highlighted in Fig. 3. It is found that these solutions are alike which shows the correctness of the present results.

## Results and Discussion

**Figure 4:**
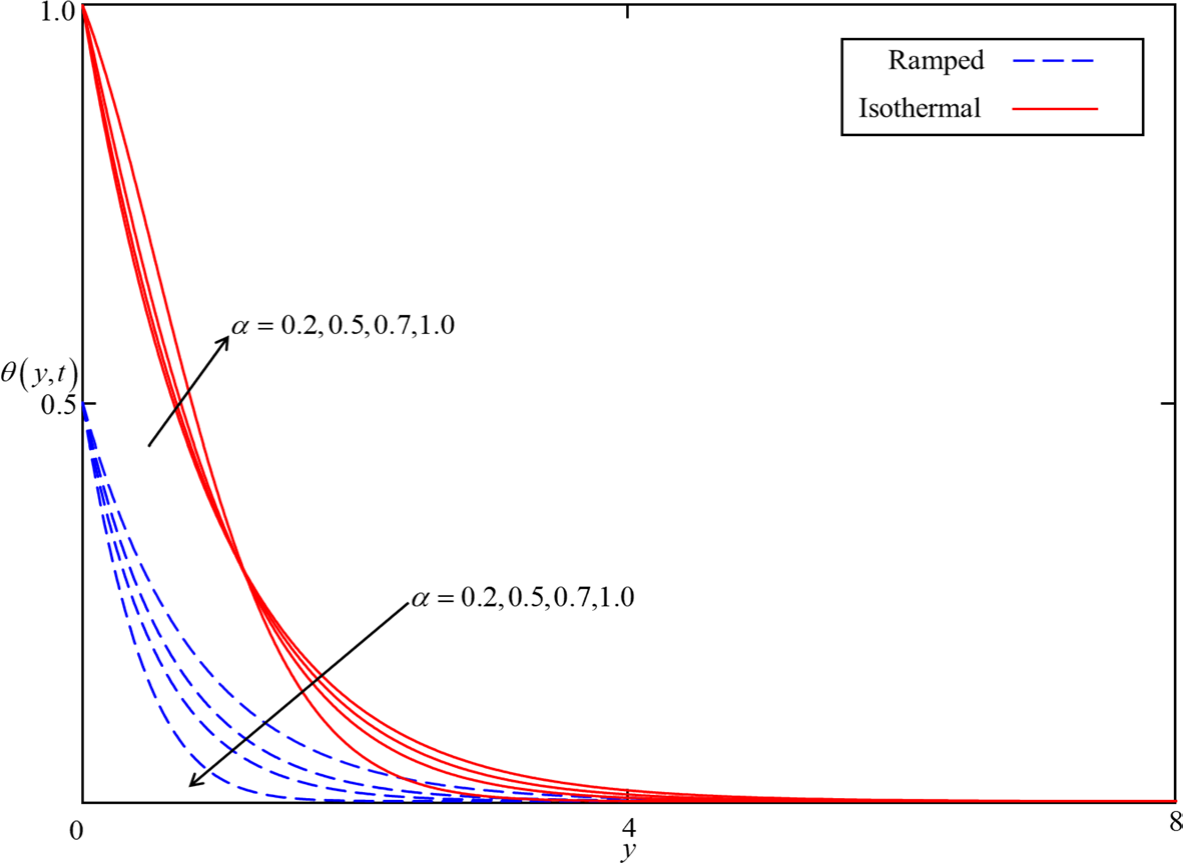
Consequence of $\alpha$ on $\theta \left( {y,t} \right)$ when $\phi = 0.04$,$Nr = 0.5$, $Q = 0.5$ in case of cylindrical shape nanoparticles.

**Figure 5:**
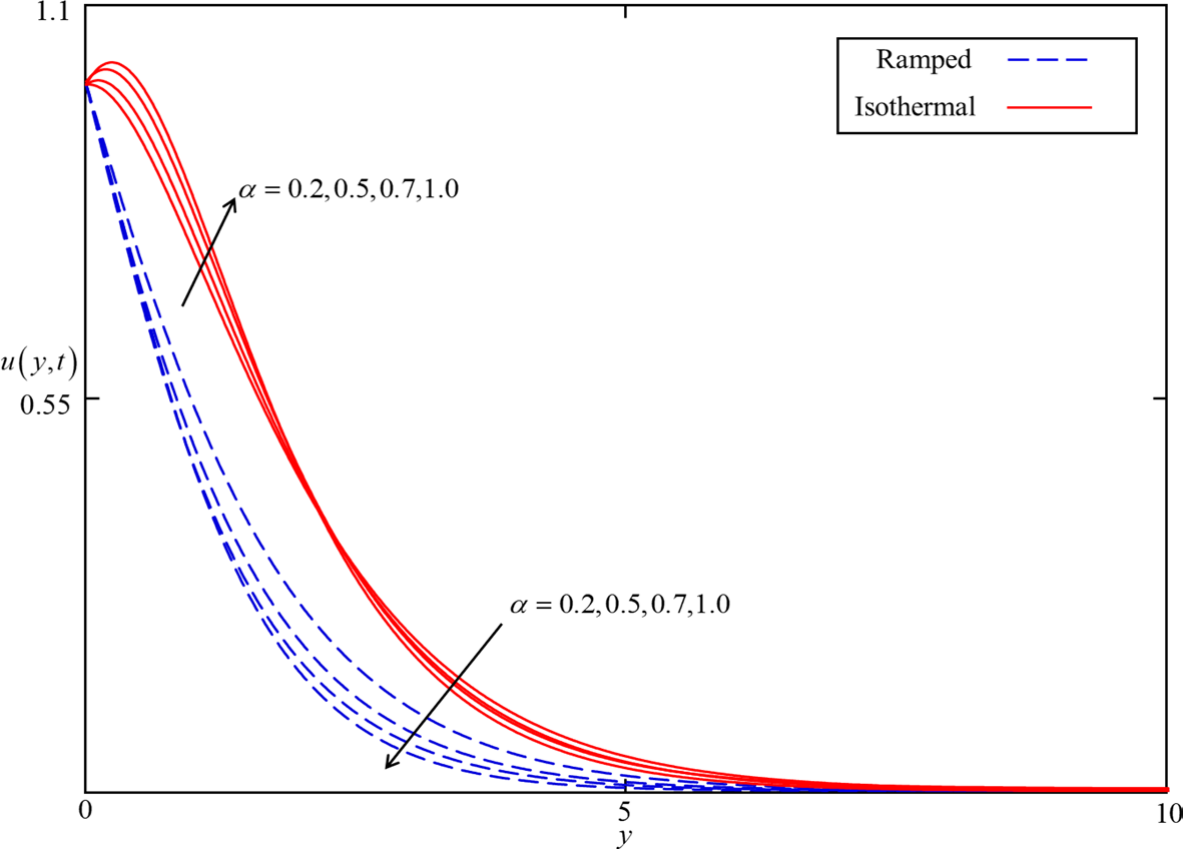
Consequence of $\alpha$ on $u\left( {y,t} \right)$ when $\phi = 0.04$, $\beta = 0.5$, $M = 0.5$, $Gr = 5$, $Nr = 0.5$, $Q = 0.5$ and $\omega t = 0.15$ in case of cylindrical shape nanoparticles.

**Figure 6:**
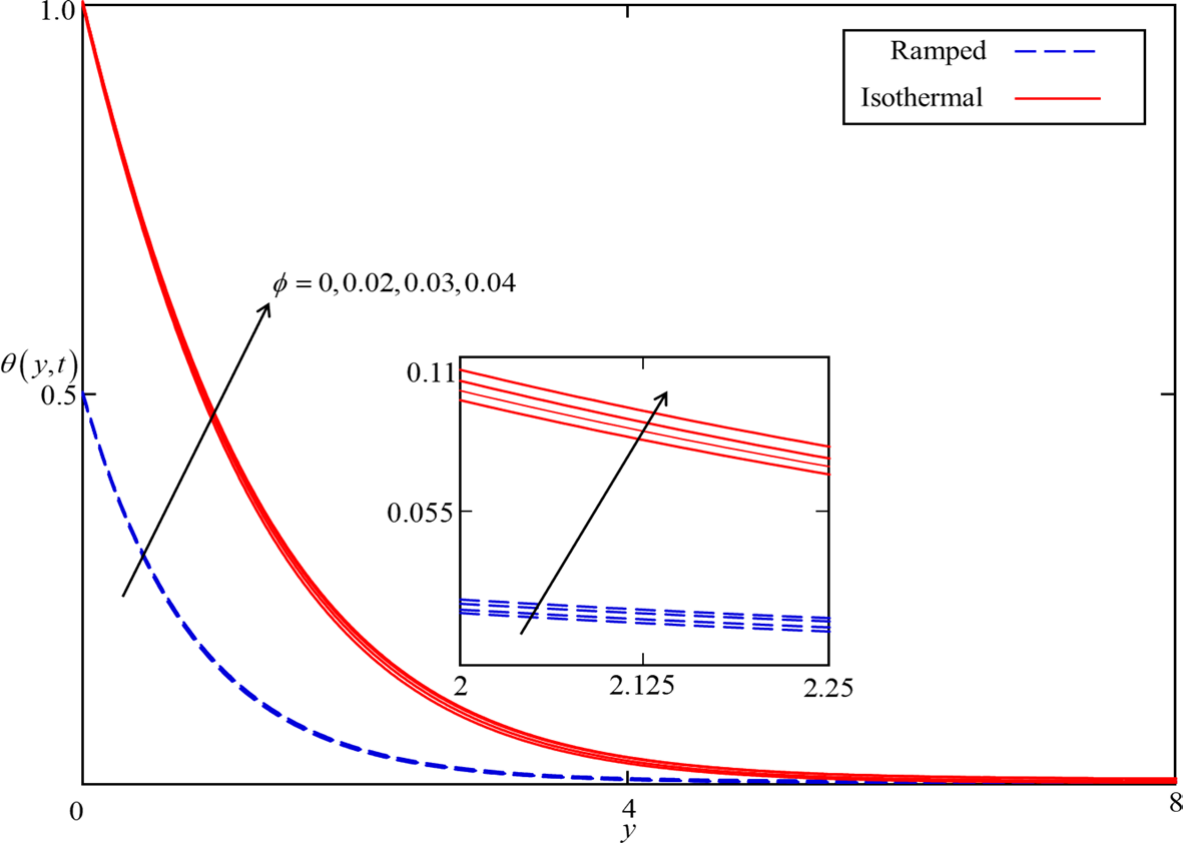
Consequence of $\phi$ on $\theta \left( {y,t} \right)$ when $\alpha = 0.5$,$Nr = 0.5$, $Q = 0.5$ in case of cylindrical shape nanoparticles.

**Figure 7:**
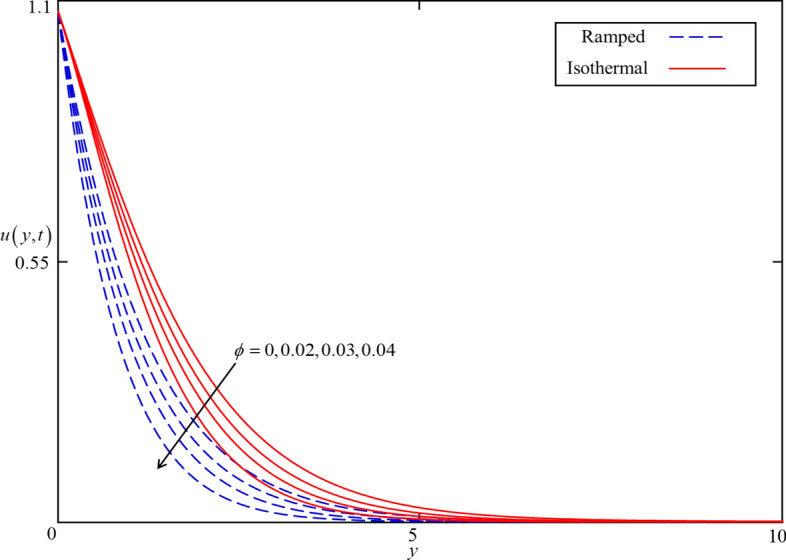
Consequence of $\phi$ on $u\left( {y,t} \right)$ when $\alpha = 0.5$, $\beta = 0.5$, $M = 0.5$, $Gr = 5$, $Nr = 0.5$, $Q = 0.5$ and $\omega t = 0.15$ in case of cylindrical shape nanoparticles.

**Figure 8:**
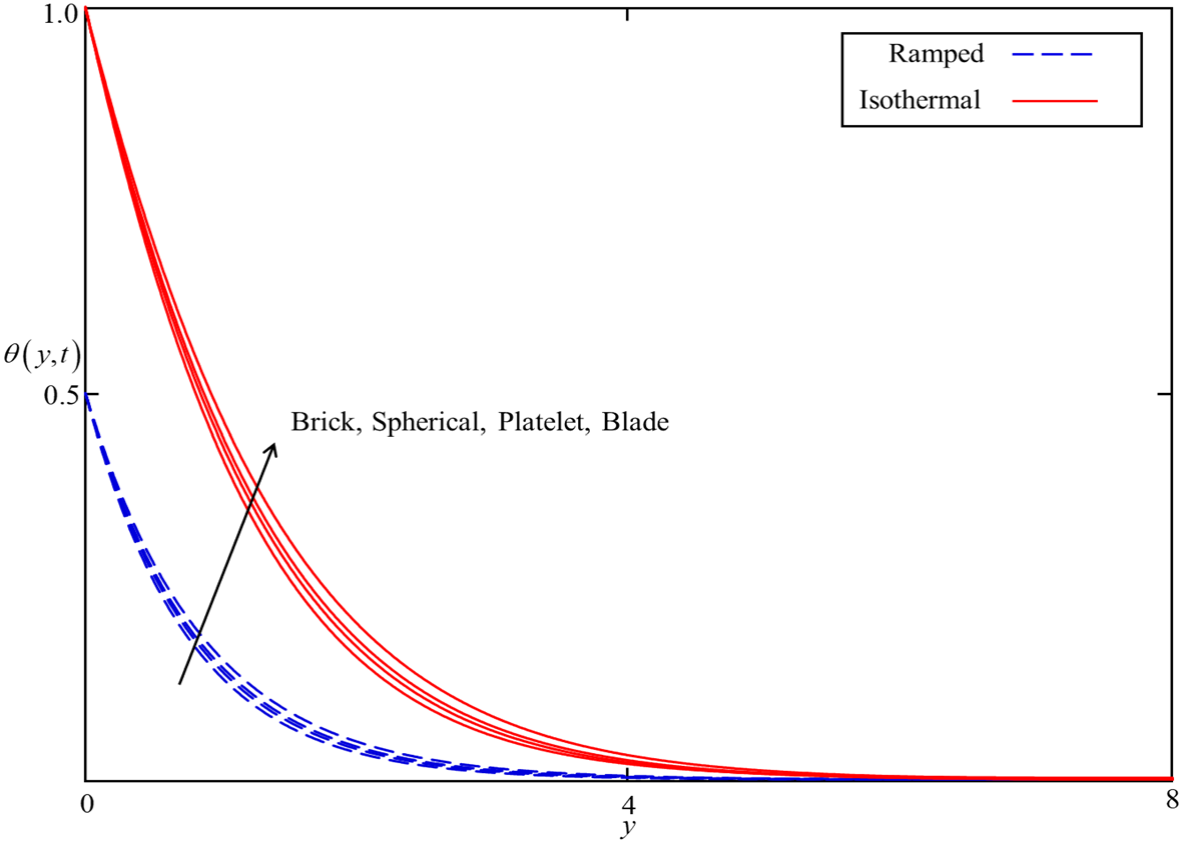
Consequence of different shapes of nanoparticles on $\theta \left( {y,t} \right)$ when $\alpha = 0.5$, $\phi = 0.04$, $Nr = 0.5$, $Q = 0.5$

**Figure 9:**
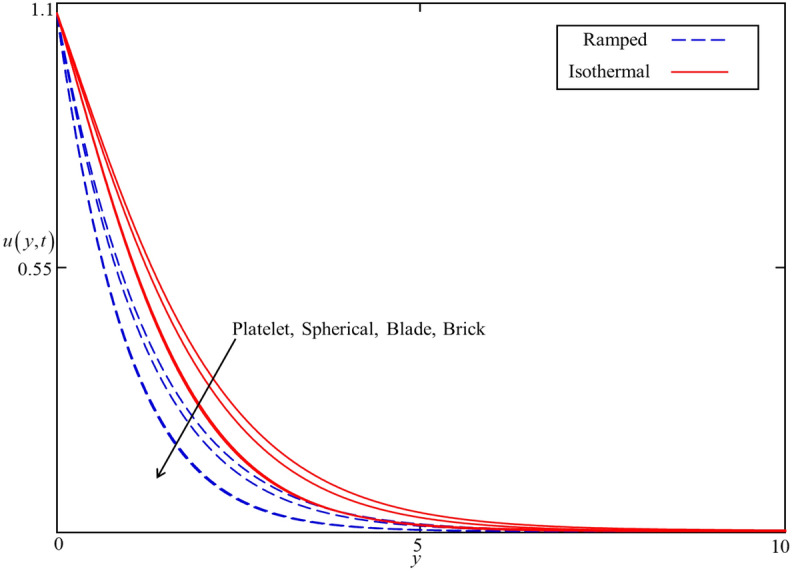
Consequence of different shapes of nanoparticles on $u\left( {y,t} \right)$ when $\alpha = 0.5$, $\phi = 0.04$, $\beta = 0.5$, $M = 0.5$, $Gr = 5$, $Nr = 0.5$, $Q = 0.5$ and $\omega t = 0.15$.

**Figure 10:**
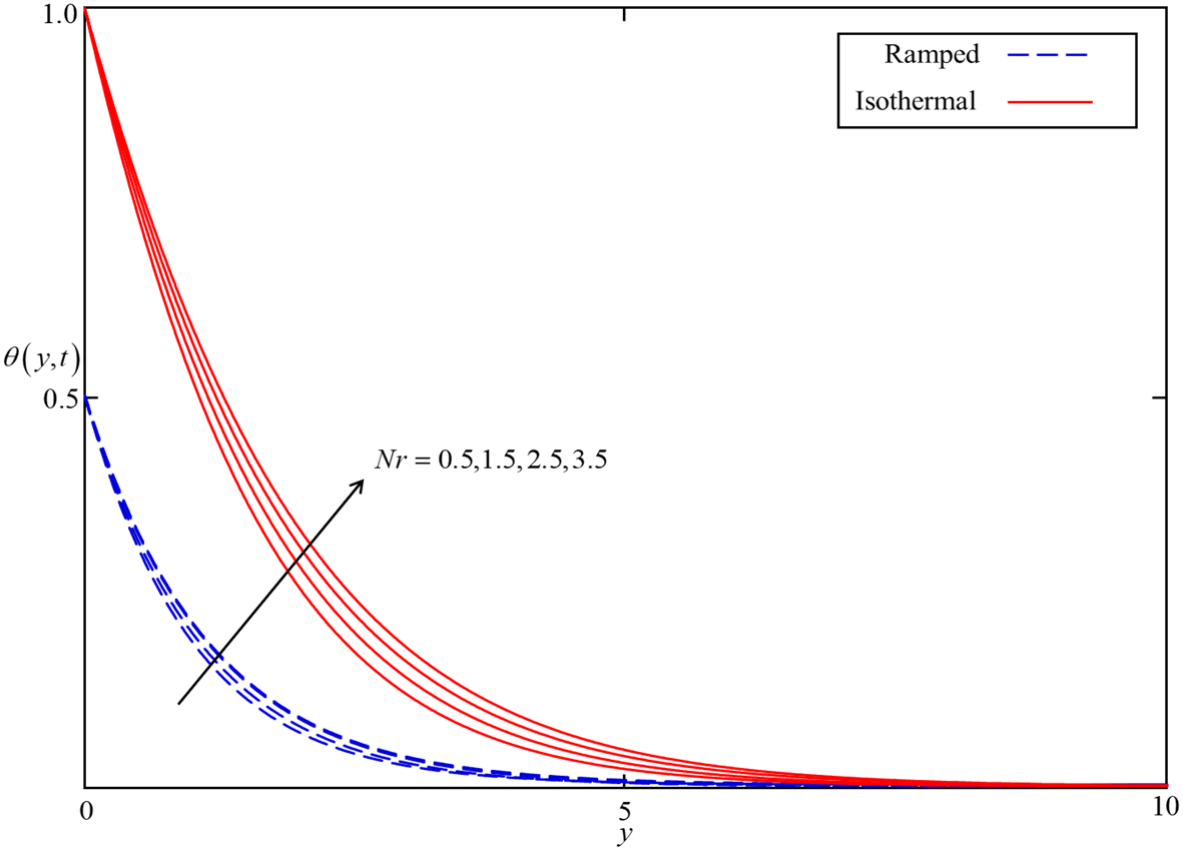
Consequence of $Nr$ on $\theta \left( {y,t} \right)$ when $\alpha = 0.5$, $\phi = 0.04$, $Q = 0.5$ in case of cylindrical shape nanoparticles.

**Figure 11:**
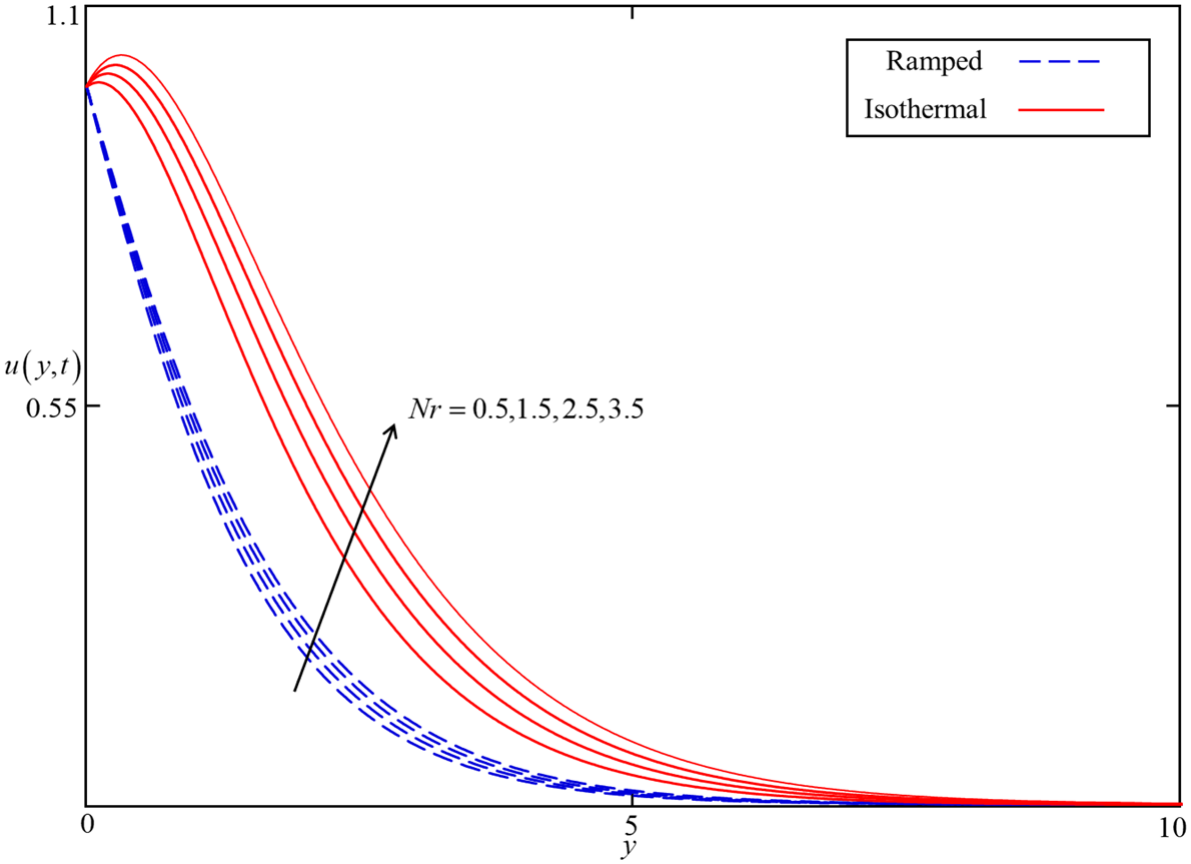
Consequence of $Nr$ on $u\left( {y,t} \right)$ when $\alpha = 0.5$, $\phi = 0.04$, $\beta = 0.5$, $M = 0.5$, $Gr = 5$, $Q = 0.5$ and $\omega t = 0.15$ in case of cylindrical shape nanoparticles.

**Figure 12:**
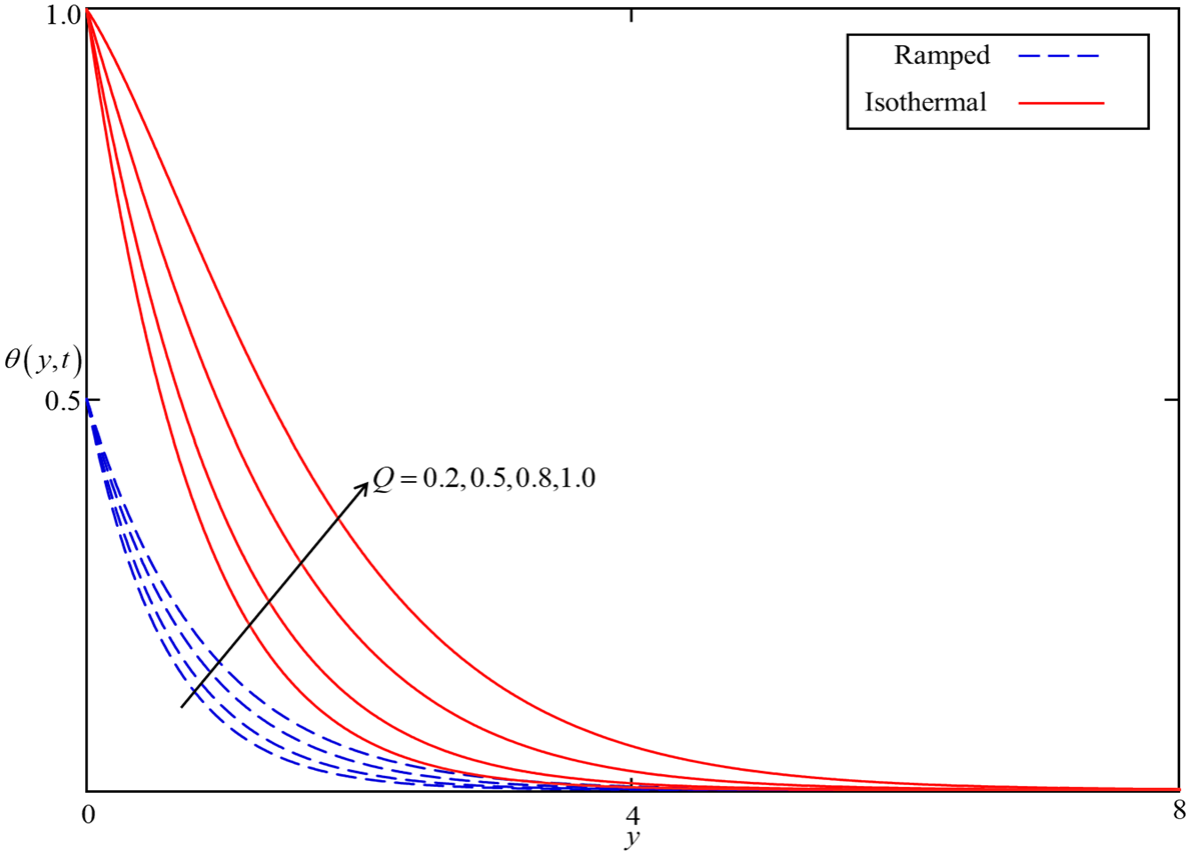
Consequence of $Q$ on $\theta \left( {y,t} \right)$ when $\alpha = 0.5$, $\phi = 0.04$, $Nr = 0.5$ in case of cylindrical shape nanoparticles.

**Figure 13:**
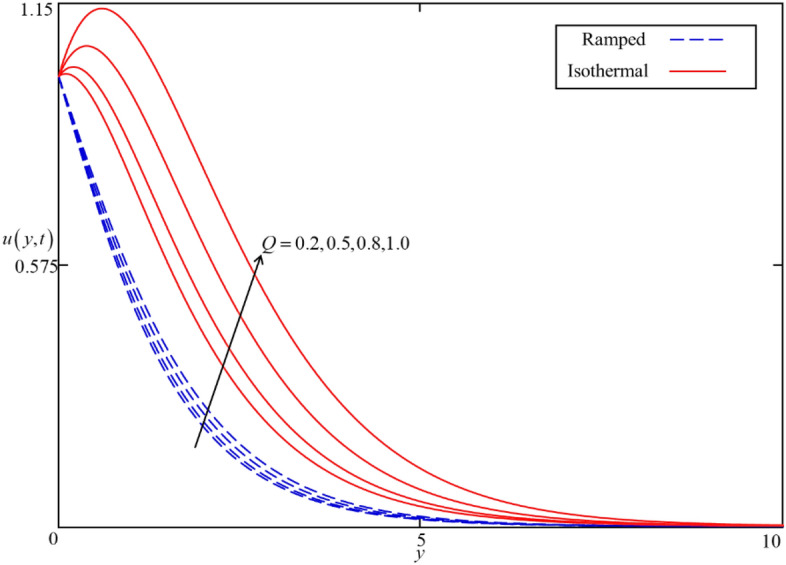
Consequence of $Q$ on $u\left( {y,t} \right)$ when $\alpha = 0.5$, $\phi = 0.04$, $\beta = 0.5$, $M = 0.5$, $Gr = 5$, $Nr = 0.5$ and $\omega t = 0.15$ in case of cylindrical shape nanoparticles.

**Figure 14:**
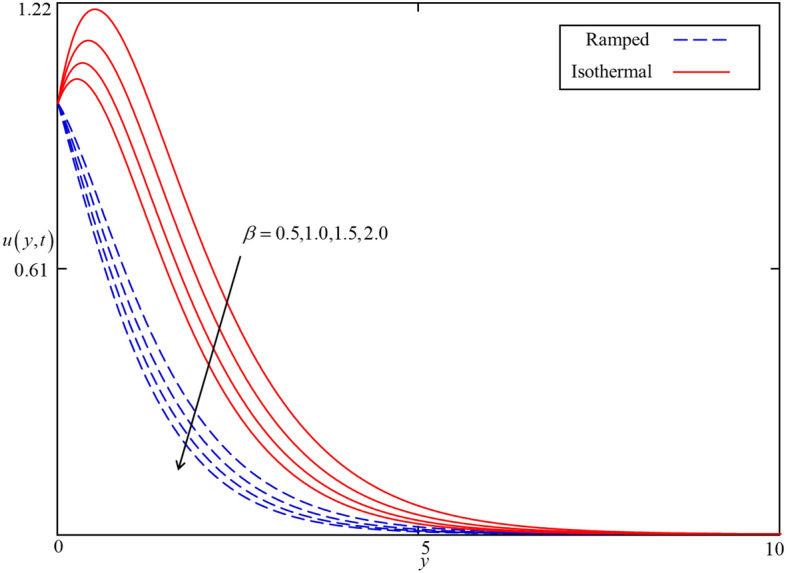
Consequence of $\beta$ on $u\left( {y,t} \right)$ when $\alpha = 0.5$, $\phi = 0.04$, $M = 0.5$, $Gr = 5$, $Nr = 0.5$, and $\omega t = 0.15$ in case of cylindrical shape nanoparticles.

**Figure 15:**
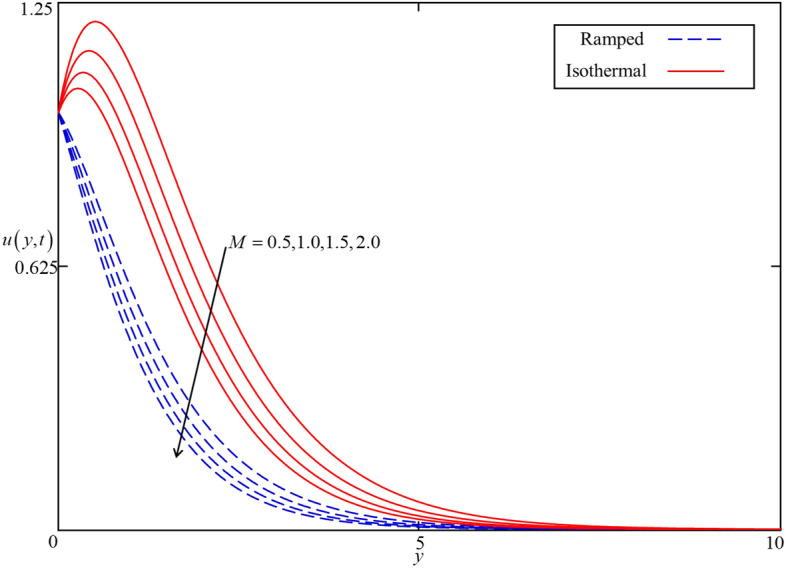
Consequence of $M$ on $u\left( {y,t} \right)$ when $\alpha = 0.5$, $\phi = 0.04$, $\beta = 0.5$, $Gr = 5$, $Nr = 0.5$, and $\omega t = 0.15$ in case of cylindrical shape nanoparticles.

**Figure 16:**
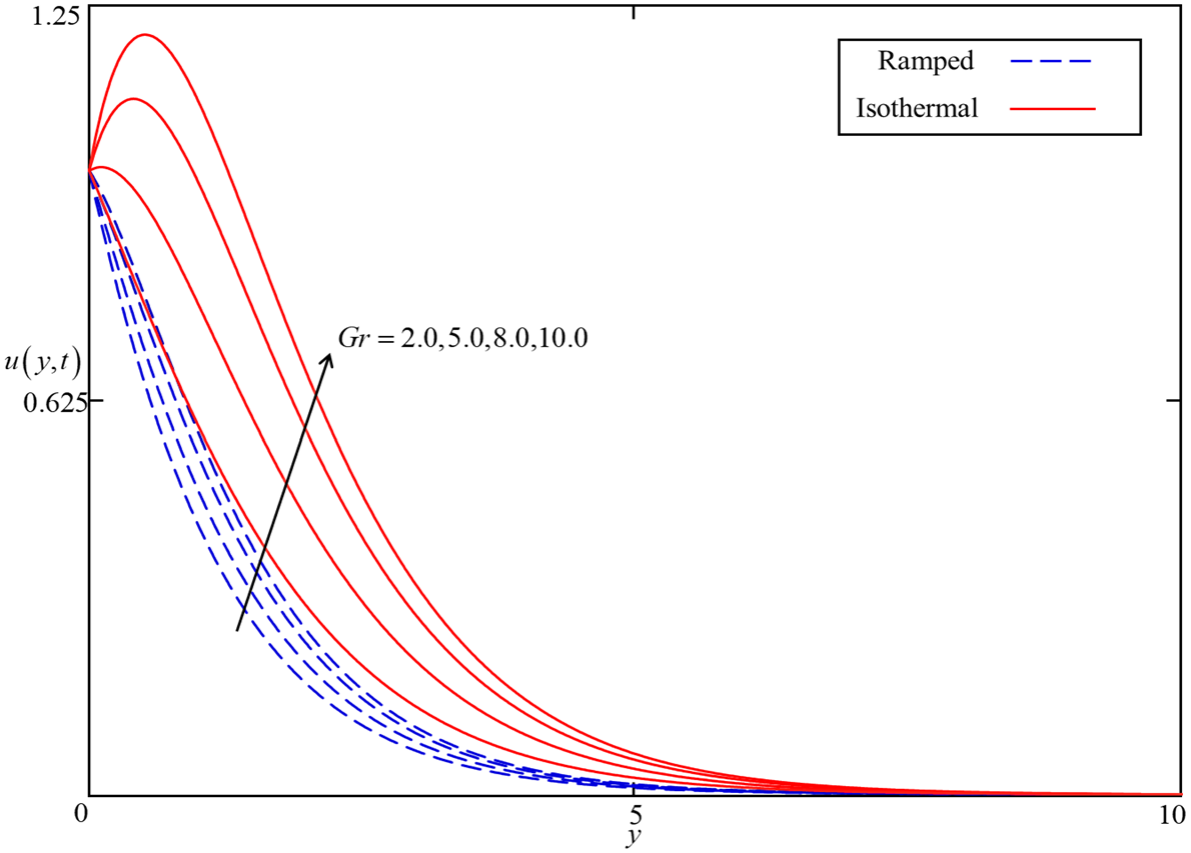
Consequence of $Gr$ on $u\left( {y,t} \right)$ when $\alpha = 0.5$, $\phi = 0.04$, $\beta = 0.5$, $M = 0.5$, $Nr = 0.5$, and $\omega t = 0.15$ for spherical s case.

The exact analytical solutions (solutions for ramped and isothermal heating) for temperature and velocity fields are computed and displayed in numerous graphs to study the impact of pertinent parameters such as fractional parameter $\alpha$, volume concentration $\phi$, shape effect of nanoparticles, thermal radiation $Nr$, heat generation $Q$, Brinkman parameter $\beta$, magnetic parameter $M$ , and thermal Grashof number $Gr$. In order to provide a clear understanding, ramped and isothermal solutions are simultaneously plotted in Figs. 4, 5, 6, 7, 8, 9, 10, 11, 12, 13, 14, 15 and 16. It is essential to underline that these graphs satisfy all the initial and boundary conditions. Moreover, for ramped heating time is chosen in $0 < t < 1$ and for isothermal heating, it is selected as $t > 0$. Precisely, for ramped heating time is chosen $t = 0.5$ and for isothermal heating, it is taken $t = 1.5$.

### Effects of flow parameters on both velocity and temperature fields

Figures 4 and 5 present the impact of $\alpha$ on temperature and velocity fields. In the case of isothermal heating, both the temperature and velocity field increase with increasing $\alpha$ near the heated plate. But this trend reverses at a certain point away from the plate. Physically this fact can be justified as $\alpha$ is increasing the thickness of thermal and momentum boundary layers gradually increasing and became thickest near the plate at $\alpha = 1$. However, away from the plate, the thermal and momentum boundary layers behave oppositely. In the case of ramped heating, the trend of temperature and velocity profile is straight forward. Increasing the value of $\alpha$, decreasing the temperature and velocity fields. This is because the thickness of thermal and momentum boundary layers is inversely related to $\alpha$ in case of ramped heating. So, when $\alpha$ is increased, thermal and momentum boundary layers are gradually decreased as a result the temperature and velocity fields decreased. Additionally, as in Figs. 6 and 7 it can be observed that the temperature and velocity field are significantly affected by $\phi$. It is found that the temperature filed increases with increasing values of $\phi$ for both ramped and isothermal heating. It can be clearly seen from Eq. (39) that an increment in $\phi$ corresponds to the enhancement in thermal conductivity of the nanofluid as a result the temperature profile increases. Furthermore, it can be seen in Fig. 4 that the velocity field decrease with increasing values of $\phi$ for both ramped and isothermal heating. This is due to the dynamic viscosity of nanofluid presented in Eq. (35). The dynamic viscosity is directly related to the volume concentration of nanoparticles. Increasing values of $\phi (0 < \phi \le 0.04)$ leads to an increase in viscosity of the nanofluid and the fluid became thick. Hence, an increase in viscosity resists to nanofluid flow.

Figures 8 and 9 depict the comparison of temperature and velocity fields for different shapes of nanoparticles. It is noticed from Fig. 8 that the temperature field for blade shape nanoparticles is higher followed by platelet, spherical, and brick shaped nanoparticles due to the shape factor $n$ involving in Eq. (39). Besides this, the velocity profile is higher for brick shape nanoparticles flowed by the blade, spherical and platelet shaped nanoparticles which depend on the values of shape constants $a$ and $b$ involving in Eq. (35). Meanwhile, the behavior of temperature and velocity fields for the thermal radiation parameter $Nr$ is studied in Figs. 10 and 11. As expected, an increase in $Nr$ results of an increase in both the temperature and velocity field as $Nr$ indicates the proportional contribution of conduction heat transfer to the thermal radiation. Hence, the temperature field signifying an increasing trend. Furthermore, increasing $Nr$ twist the rate of heat transfer to the nanofluid as a result the attractive forces holding the nanofluid molecule weaken as a result, decreasing the viscosity which accelerates the fluid velocity. Variations in temperature and velocity fields due to heat generation $Q$ are depicted in Figs. 12 and 13, where $Q$ is selected arbitrary 0.2, 0.5, 0.8, and 1.0. It is observed that both the temperature and velocity fields for ramped and isothermal heating increasing for increasing values of $Q$ because the existence of heat generation causes an increment in the energy level due to which the thickness of thermal and momentum boundary grow at the oscillating boundary as a result the temperature and velocity field increases.

### The impact of flow parameters which effect only the velocity field

The influence of the Brinkman type fluid parameter $\beta$ on the velocity profiles for isothermal and ramped heating is displayed in Fig. 14. $\beta$ is the magnitude of the drag force of a highly non-Darcy’s porous medium. The velocity fields for both isothermal and ramped heating decelerated with an increment in $\beta$ because of a strong drag force. Hence, increment in $\beta$ increase the drag forces which decelerate the velocity field. Meanwhile, the effect of the magnetic parameter $M$ is illustrated in Fig. 15 on the ramped and isothermal velocity fields. It is revealed that the isothermal velocity is higher than the ramped velocity. The isothermal and ramped velocity fields decelerated together for greater values of $M$ due to the applied magnetic field which results in the presence of intense Lorentz force. This force works as a dragging force exhibits persistent resistance to the nanofluid flow. Ultimately, the isothermal and ramped velocity filed dropped. But away from the plate, the Lorentz force became poor and nanofluid comes to rest. Besides this, the influence of thermal Grashof number $Gr$ is highlighted in Fig. 16 for both ramped and isothermal heating. It is demonstrated in this figure that the velocity field increases with increasing $Gr$. The $Gr$ shows the proportional strength of the buoyancy force to the viscous force. thereby, an increase in $Gr$ leads to an increase in thermal buoyancy force. In the proposed problem, the convection flow of nanofluid driven by thermal buoyancy force is considered. As a result, it has a tendency to increase the velocity field in both ramped and isothermal heating cases.

## Conclusion

This manuscript has been considered the MHD flow of Ferro-nanofluid near a vertical plate in the presence of thermal radiation, heat generation, and the shape effect of the nanoparticle. The oscillating boundary conditions together with isothermal and ramped heating have been taken at the solid boundary. The flow phenomenon has been modeled in the form of time-fractional Caputo-Fabrizio fractional derivatives. The model has been solved for the exact analytical solutions via the Laplace transform method. The obtained solutions for temperature and velocity field have been simultaneously plotted for ramped and isothermal heating. The results have been revealed that the temperature field for blade shape nanoparticles is higher followed by platelet, spherical and brick shaped nanoparticles due to shape factor $n$ whereas the velocity profile is higher for brick shape nanoparticles flowed by the blade, spherical and platelet shaped nanoparticles which depend on the values of shape constants $a$ and $b$. Moreover, the temperature and velocity fields increase with increasing values of $\alpha$ near the plate in case of isothermal heating. But away from the plate, this effect reverses. Besides this the temperature field increase with increasing $\phi$. However, the velocity filed behaves opposite to this for $\phi$. Meanwhile, the temperature and velocity fields increase with increasing $Nr$ and $Q$. Finally, it has been noticed that the velocity field decreases for increasing $\beta$ and $M$ whereas it increases with increasing $Gr$. Furthermore, this work can be extended in the future by developing a non-linear model with fractional derivatives. Meanwhile, the fractional boundary layer flow can be taken in a channel and cylindrical tubes with new fractional operators (Supplementary information S1).

## Supplementary Information

Supplementary Information
