# Supplementary material for: Shape effect on MHD flow of time fractional Ferro-Brinkman type nanofluid with ramped heating
Source: Sci Rep. 2021 Feb 12;11:3725. doi: 10.1038/s41598-020-78421-z (PMC7881191; doi:10.1038/s41598-020-78421-z)
Supplement: Supplementary file 1 — Supplementary Information [file 41598_2020_78421_MOESM1_ESM.docx]

**Shape Effect on MHD Flow of Time Fractional Ferro-Brinkman Type Nanofluid with Ramped Heating**

**Muhammad Saqib^1^, Ilyas Khan^2^, Sharidan Shafie^1^, and Ahmad Qushairi Mohamad^1^**

^1^Department of Mathematical Sciences, Faculty of Science, Universiti Teknologi Malaysia JB, 81310 Johor Bahru, Johor Malaysia.

^2^Department of Mathematics, College of Science Al-Zulfi, Majmaah University, Al-Majmaah 11952, Saudi Arabia.

**Corresponding authors: Ilyas khan:* [*i.said@mu.edu.sa*](mailto:i.said@mu.edu.sa)*, Sharidan Shafie:* [*sharidan@utm.my*](mailto:sharidan@utm.my)

**Appendix-A**

. (1A)

. (2A)

. (3A)

(4A)

where for $c\neq0$,$a_{1}=\frac{a}{c}, a_{2}=\frac{bc-ad}{c^{2}}, a_{3}=\frac{d}{c}$,
